# Transcriptomic and Metabolomic Insights into Tissue- and Cultivar-Dependent Polysaccharide Accumulation in *Anoectochilus roxburghii*

**DOI:** 10.3390/ijms27156917

**Published:** 2026-08-01

**Authors:** Xiaoqun Peng, Chao Liu, Yiping Liu, Haiwei Xie, Liangliang He, Yan Xie, Zhisheng Zhang, Menglong Wang, Shu Chen

**Affiliations:** 1 School of Life Sciences, Huizhou University, Huizhou 516007, China; xiaoqunpeng@hzu.edu.cn (X.P.); lyp15907539539@foxmail.com (Y.L.); xiehaiwei324@163.com (H.X.); 2 Department of Grass Science, College of Forestry and Landscape Architecture, South China Agricultural University, Guangzhou 510642, China; chaoliu@stu.scau.edu.cn (C.L.); liangl_20@stu.scau.edu.cn (L.H.); yanxieovo@stu.scau.edu.cn (Y.X.); zszhang@scau.edu.cn (Z.Z.); 3 South China Agricultural University and Zhongshan Joint Innovation Center, Zhongshan 528478, China

**Keywords:** *Anoectochilus roxburghii*, polysaccharide accumulation, transcriptomics, sugar metabolomics, carbohydrate metabolism

## Abstract

Polysaccharides are major bioactive constituents and quality-related components of *Anoectochilus roxburghii*. However, how polysaccharide accumulation varies among cultivars and tissues remains poorly understood. Here, crude polysaccharide content was quantified in leaves and stems of three cultivars, Yuanye (YY), Hongxia (HX), and Jianye (JY), and the underlying molecular basis was investigated using transcriptomics, targeted sugar metabolomics, and integrated transcriptome–metabolome analysis. Stems accumulated significantly higher levels of crude polysaccharides than leaves, whereas cultivar-dependent differences were most pronounced in stems. JY stems showed the highest crude polysaccharide content, reaching 357.81 mg g^−1^ dry weight. Transcriptomic and metabolomic profiles clearly differentiated the cultivars and tissues. Metabolic differences were concentrated at a limited number of sugar-metabolic nodes, whereas the transcriptome showed extensive transcriptional reprogramming. Integrative analyses consistently implicated starch and sucrose metabolism, precursor-sugar interconversion, nucleotide-sugar biosynthesis, and sugar transport, partitioning, and intracellular allocation in differential polysaccharide accumulation. Glucose and D-fructose emerged as key hub metabolites linking transcriptional variation to sugar-metabolic remodeling. These results suggest that tissue- and cultivar-dependent polysaccharide accumulation is associated with coordinated changes in sucrose turnover, precursor sugar supply, nucleotide-sugar formation, and sugar partitioning, particularly in stems. This study provides molecular evidence for the selection of high-polysaccharide germplasm and quality evaluation of *A. roxburghii*.

## 1. Introduction

*Anoectochilus roxburghii*, a perennial herb belonging to the genus *Anoectochilus* in the family Orchidaceae, is one of the traditional rare Chinese medicinal plants and possesses both medicinal and edible value. It has long been used in folk medicine for clearing heat, cooling blood, eliminating dampness, detoxifying, and promoting general health. Modern pharmacological studies have further demonstrated that *A. roxburghii* exhibits multiple biological activities, including hypoglycemic, antioxidant, anti-inflammatory, hepatoprotective, and immunomodulatory effects [1]. With the increasing exploitation and utilization of medicinal and edible homologous resources, *A. roxburghii* has become an important specialty medicinal plant with considerable economic value and industrial potential.

Polysaccharides are major bioactive components of *A. roxburghii* and serve as key quality indicators. Previous studies have shown that *A. roxburghii* polysaccharides possess significant hypoglycemic, antioxidant, and lipid-lowering activities, and their bioactivities are closely related to monosaccharide composition, glycosidic linkage type, molecular weight, and spatial conformation [2,3]. Recent evidence further demonstrated that *A. roxburghii* polysaccharides could alleviate DSS-induced ulcerative colitis by restoring intestinal barrier integrity, suppressing MAPK/NF-κB-mediated inflammatory responses, and modulating gut microbiota, highlighting their potential as functional food and medicinal quality-related components [4]. In addition, *A. roxburghii* polysaccharides exhibit considerable structural heterogeneity, with different fractions differing in both chemical composition and higher-order structure [5]. However, existing studies have mainly focused on extraction and purification, structural characterization, and bioactivity evaluation, whereas knowledge regarding differences in polysaccharide accumulation among cultivars and tissues, as well as the underlying mechanisms, remains limited.

The accumulation of bioactive compounds in plants is influenced by genetic background and organ differentiation, often showing clear cultivar- and tissue-specific patterns. In medicinal plants, different tissues play distinct roles in photosynthetic assimilation, material transport, and storage, often resulting in substantial differences in carbohydrate metabolism and bioactive compound accumulation patterns. At the same time, different cultivars may develop distinct quality characteristics owing to differences in carbon partitioning, precursor sugar supply, and metabolic regulatory capacity. In *A. roxburghii*, systematic studies on differences in polysaccharide accumulation among cultivars and tissues are still scarce, particularly those integrating sugar metabolic reprogramming and transcriptional regulation to elucidate their molecular basis.

In recent years, integrated transcriptomic and metabolomic analyses have become powerful approaches for dissecting the formation mechanisms of plant bioactive compounds. Transcriptomics enables the systematic identification of candidate genes associated with specific metabolic processes, whereas metabolomics directly reflects metabolite accumulation states and changes at key metabolic nodes. Their integration facilitates the construction of “gene–metabolite–trait” association networks and provides a more comprehensive understanding of the molecular basis underlying complex traits. Previous studies on *A. roxburghii* polysaccharides have mainly focused on structural characterization and pharmacological activity, while genome-wide insights into the molecular basis of cultivar- and tissue-specific polysaccharide accumulation remain largely lacking. To address this gap, the present study employed three *A. roxburghii* cultivars—Hongxia (HX), Jianye (JY), and Yuanye (YY)—to systematically compare polysaccharide content in leaves and stems, and combined transcriptome sequencing with targeted sugar metabolomics to investigate differences in polysaccharide accumulation among cultivars and tissues and their potential molecular regulatory mechanisms. The results provide a theoretical basis for elite germplasm screening, medicinal quality evaluation, and precision cultivation of *A. roxburghii*.

Cultivated *A. roxburghii* accessions have been reported to exhibit abundant morphological diversity in plant height, leaf color, leaf size, and vein pattern, and recent transcriptomic analysis has identified cultivar-specific expression patterns and EST-SSR markers useful for diversity assessment [6]. These findings indicate that cultivated *A. roxburghii* materials may differ not only in external morphology but also in genetic background and metabolic regulation. The three cultivars used in this study, HX, JY, and YY, are commercially cultivated *A. roxburghii* materials widely used in local production. They differ in stable morphological and growth-related traits: YY is characterized by relatively round leaves, HX by reddish leaf/stem pigmentation, and JY by relatively narrow and pointed leaves. These materials are maintained mainly through clonal propagation or tissue culture, which helps preserve cultivar-specific phenotypes. Although their detailed breeding pedigrees and domestication histories are not fully documented, their stable morphological differences suggest distinct genetic backgrounds. Because cultivated medicinal plants are often selected for growth vigor, morphology, adaptability, biomass, and medicinal quality, these cultivars may also differ in carbon allocation and carbohydrate metabolism. Therefore, HX, JY, and YY provide useful materials for evaluating whether cultivar-dependent metabolic divergence is associated with polysaccharide accumulation in *A. roxburghii*.

## 2. Results

### 2.1. Polysaccharide Accumulation Differs Significantly Among Cultivars and Tissues of A. roxburghii

**Figure 1:**
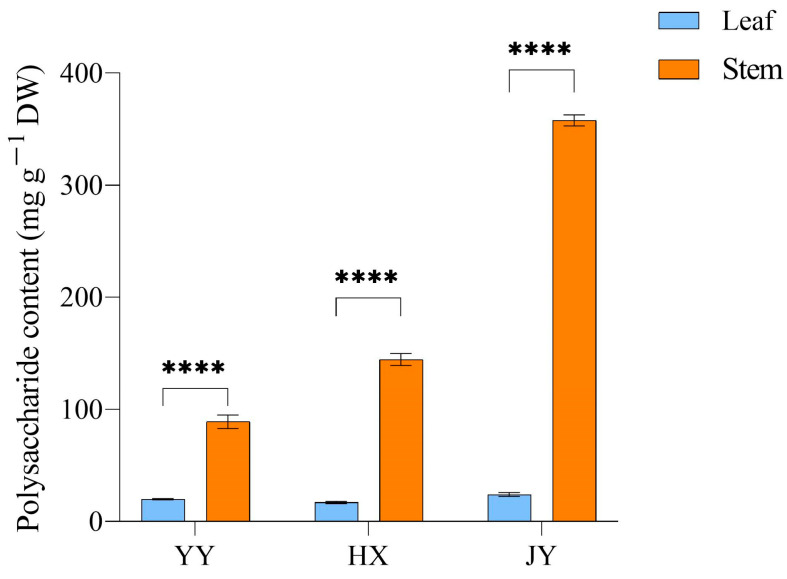
Crude polysaccharide contents in leaves and stems of three *A. roxburghii* cultivars. Bars represent the mean ± SD of three biological replicates, and polysaccharide content is expressed as mg g^−1^ DW. **** indicates *p* < 0.0001. Statistical significance was evaluated using two-way ANOVA followed by Šidák’s multiple comparisons test for leaf–stem comparisons within the same cultivar and Tukey’s multiple comparisons test for cultivar comparisons within the same tissue. Detailed ANOVA results, adjusted *p* values, variance components, and assumption-test results are provided in Supplementary Table S2.

To evaluate differences in crude polysaccharide accumulation among cultivars and tissues of *A. roxburghii*, polysaccharide contents were determined in leaves and stems of YY, HX, and JY (Figure 1; Supplementary Table S1). Before two-way ANOVA, normality of residuals and homogeneity of variance were checked, and no major violations of ANOVA assumptions were detected (Supplementary Table S2). Two-way ANOVA showed that cultivar, tissue, and their interaction significantly affected crude polysaccharide content, with tissue explaining the largest proportion of total variance (Supplementary Table S2).

Across all three cultivars, stems accumulated significantly more crude polysaccharides than leaves. Among cultivars, leaf polysaccharide content showed relatively modest variation, whereas stem polysaccharide content differed markedly among cultivars. JY stems showed the highest crude polysaccharide content among all samples, indicating a stronger polysaccharide accumulation capacity in this cultivar, particularly in stem tissue (Figure 1; Supplementary Tables S1 and S2).

Overall, crude polysaccharide accumulation in *A. roxburghii* was influenced by both tissue background and genetic background. Tissue type exerted the dominant effect, while cultivar-dependent differences were mainly manifested in stems.

### 2.2. Global Transcriptomic and Metabolomic Profiles Reveal Clear Cultivar- and Tissue-Separation Patterns

**Figure 2:**
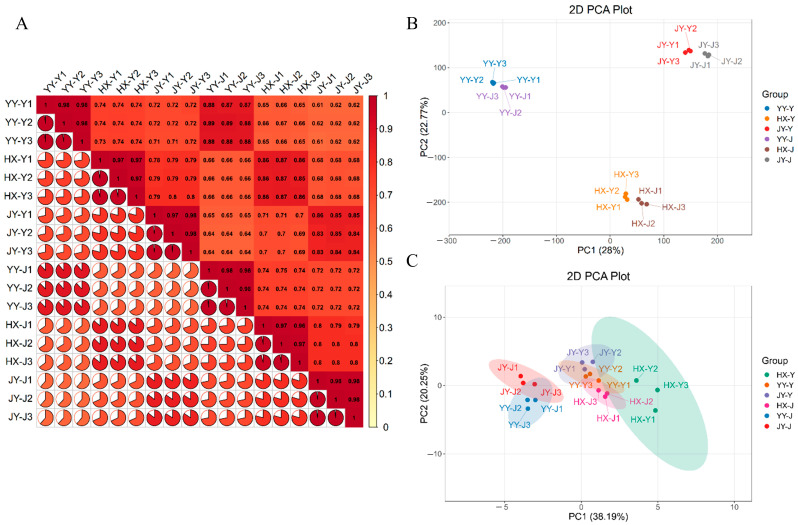
Global distribution patterns of transcriptomic and metabolomic profiles among different cultivars and tissues of *A. roxburghii*. (**A**) Pearson correlation heatmap among the 18 transcriptomic samples; (**B**) principal component analysis (PCA) of transcriptomic samples; (**C**) PCA of metabolomic samples. Each point represents one biological replicate. YY, HX, and JY denote the Yuanye, Hongxia, and Jianye cultivars, respectively; Y and J denote leaf and stem, respectively. Ellipses indicate the clustering range of each sample group in PCA space.

To assess the overall molecular differences among cultivars and tissues, Pearson correlation analysis and principal component analysis (PCA) were performed separately on transcriptomic and metabolomic data from the 18 samples (Figure 2).

The Pearson correlation heatmap showed high consistency among biological replicates within each group, indicating good reproducibility of the transcriptomic data (Figure 2A). Samples from the same cultivar generally showed higher similarity than samples from different cultivars, suggesting a clear cultivar-dependent transcriptomic background.

Transcriptomic PCA further revealed clear separation among cultivars, with biological replicates clustering closely within each group, further supporting data stability (Figure 2B). Along PC1, YY samples were generally located on the left side of the plot, whereas HX and JY samples were mainly distributed on the right side. Along PC2, HX samples were mainly located in the negative region, whereas JY samples were mainly located in the positive region. These results indicate clear global transcriptomic divergence among the three cultivars, with the difference between YY and HX/JY mainly captured by PC1, and the distinction between HX and JY further reflected by PC2. In addition, leaves and stems within each cultivar also showed a certain degree of separation, suggesting that tissue type also contributed to shaping global gene expression patterns.

Metabolomic PCA also showed distinct sample distribution patterns associated with both cultivar and tissue effects (Figure 2C). Biological replicates generally clustered well, although the degree of dispersion varied among groups, with the HX-Y group showing relatively greater within-group variation. Overall, metabolomic samples exhibited both cultivar and tissue effects in PCA space. HX leaf and stem samples were mainly located in the positive region of PC1, whereas the separation between leaf and stem samples was more evident in YY and JY.

Taken together, both transcriptomic and metabolomic analyses indicated stable global molecular differences among cultivars and tissues. However, the major separation patterns reflected by the two omics datasets were not completely identical: cultivar divergence was more pronounced in the transcriptome, whereas the metabolome simultaneously showed relatively clear cultivar differences and tissue separation within certain cultivars. These results provide an overall basis for subsequent analyses of DEGs, DAMs, and their integrative relationships.

### 2.3. Key Metabolic Changes and Extensive Transcriptional Reprogramming Are Jointly Associated with Differential Polysaccharide Accumulation

To further elucidate the molecular basis underlying differences in polysaccharide accumulation among cultivars and tissues, sugar-related differentially accumulated metabolites (DAMs) and differentially expressed genes (DEGs) were systematically analyzed across the nine pairwise comparison groups. Because the targeted sugar metabolomic dataset mainly represented free sugars and related small-molecule intermediates, these analyses were used to characterize sugar metabolic redistribution associated with crude polysaccharide accumulation rather than the final polymer composition.

For pairwise metabolite comparisons, sugar-related DAMs were screened using the criteria of fold change (FC) ≥ 2 or FC ≤ 0.5. Overall, only a limited number of sugar-related DAMs were identified in each comparison, indicating that metabolite-level variation was concentrated at a small set of sugar metabolic nodes. Frequency analysis showed that D-fructose, D-xylose, glucose, maltose, inositol, and D-arabinose were the most recurrent DAMs across comparisons (Table 1; Supplementary Table S3). These metabolites are mainly associated with sucrose turnover, hexose partitioning, pentose-related metabolism, and sugar alcohol metabolism.

The direction of DAM changes revealed both tissue- and cultivar-dependent sugar metabolic redistribution. In tissue comparisons, stems generally showed increased accumulation of several hexose- and pentose-related metabolites, particularly in JY and YY, whereas HX showed fewer significant sugar metabolic changes. Specifically, multiple metabolites, including glucose, D-fructose, D-xylose, inositol, and maltose, were increased in stems in both JY-J vs. JY-Y and YY-J vs. YY-Y, while only D-fructose and D-xylose were increased in stems in HX-J vs. HX-Y. In cultivar comparisons, the altered metabolites mainly involved glucose, D-fructose, D-xylose, maltose, sucrose, sugar alcohols, and amino sugar-related compounds, but the specific combinations differed between leaves and stems. Detailed metabolite identities, change directions, and fold-change values for each comparison are provided in Supplementary Table S3.

**Figure 3:**
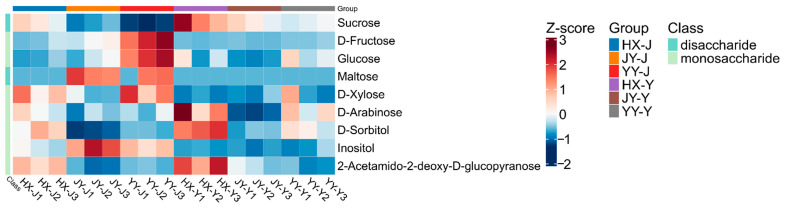
Accumulation patterns of representative sugar-related metabolites among different cultivars and tissues of *A. roxburghii*. Heatmap showing the relative accumulation of nine representative sugar-related metabolites among different cultivars and tissues. Colors indicate row-scaled Z-scores, with red and blue representing relatively high and low accumulation, respectively. The metabolites shown include high-frequency DAMs repeatedly detected in multiple pairwise comparisons, as well as representative sugar metabolic nodes illustrating the major tissue and cultivar differentiation patterns.

To visualize the overall accumulation patterns of representative sugar-related metabolites, nine metabolites were selected for heatmap analysis (Figure 3). These metabolites included recurrent DAMs and representative sugar metabolic nodes that reflected major tissue- and cultivar-dependent differences. The heatmap showed clear separation of metabolite accumulation patterns among tissues and cultivars. In general, D-fructose, glucose, and maltose tended to accumulate at relatively higher levels in some stem samples, with D-fructose particularly prominent in YY-J and maltose relatively enriched in JY-J and YY-J. By contrast, sucrose displayed an opposite or cultivar-biased pattern, showing relatively higher accumulation in HX-Y but lower levels in JY-J and YY-J. Pentose-related metabolites and sugar alcohol-related compounds also showed distinct tissue- or cultivar-associated distribution patterns. For example, D-xylose was relatively elevated in several stem samples, whereas inositol was relatively enriched in JY stems. The accumulation patterns of all detected sugar-related metabolites are shown in Supplementary Figure S1, and detailed quantitative changes are provided in Supplementary Table S3.

In addition to pairwise comparisons, multi-group differential metabolite analysis was performed among the three cultivars separately within stem and leaf tissues using the criteria of VIP > 1 and ANOVA *p* < 0.05. A total of 10 candidate differential metabolites were identified in stems, whereas only 5 were identified in leaves (Supplementary Table S4). The larger number of candidate differential metabolites identified in stems than in leaves is consistent with the more pronounced cultivar-dependent differences in crude polysaccharide accumulation observed in stem tissues.

In contrast to the limited number of sugar-related DAMs, transcriptomic analysis revealed extensive transcriptional reprogramming across the comparison groups. DEGs were identified using DESeq2 with Benjamini–Hochberg false discovery rate (FDR) correction, and genes satisfying |log_2_FC| ≥ 1 and FDR < 0.05 were considered differentially expressed. Tissue comparisons within cultivars showed that 9.91–12.06% of annotated genes were upregulated and 7.53–9.17% were downregulated. In contrast, cultivar comparisons within the same tissue generally produced broader transcriptomic divergence, with 16.00–17.61% and 16.00–17.54% of genes upregulated in stems and leaves, respectively, and 14.97–21.78% and 15.39–21.31% downregulated in stems and leaves, respectively. These results suggest that cultivar-specific genetic background exerted a stronger influence on global gene expression patterns than tissue differentiation. Detailed DEG numbers, up- and downregulated gene counts, and proportions for each comparison are provided in Supplementary Table S5.

Taken together, these results indicate that differential polysaccharide accumulation in *A. roxburghii* is associated with two complementary molecular features: metabolite changes concentrated at a limited number of key sugar metabolic nodes and extensive transcriptomic reprogramming involving a large number of genes. This pattern suggests that polysaccharide accumulation differences among cultivars and tissues are likely shaped by coordinated regulation of carbohydrate metabolism rather than by changes in a single metabolite or pathway.

### 2.4. Integrated KEGG Analysis Indicates That Carbohydrate Metabolism Is the Major Metabolic Basis of Differential Polysaccharide Accumulation

To further dissect the metabolic basis underlying differences in polysaccharide accumulation among cultivars and tissues, KEGG enrichment analyses were performed separately for DEGs and DAMs in the nine pairwise comparison groups. Pathways to which both DEGs and DAMs were mapped within the same comparison were defined as shared KEGG pathways. For each recurrent shared pathway, the numbers of pathway-mapped DEGs and DAMs, together with Benjamini–Hochberg-adjusted *p* values from DEG-based and DAM-based KEGG enrichment analyses, were summarized in Table 2 and Supplementary Table S6B. Recurrently detected shared pathways across all comparisons are listed in Supplementary Table S6A and Figure S2.

Among all shared pathways, starch and sucrose metabolism, amino sugar and nucleotide sugar metabolism, biosynthesis of nucleotide sugars, fructose and mannose metabolism, and galactose metabolism were recurrently detected across comparisons (Table 2; Supplementary Table S6). These pathways mainly involve sucrose utilization, interconversion among sugar components, and the formation of activated sugar donors such as UDP- and GDP-sugars.

Beyond these core pathways, pentose and glucuronate interconversions (ko00040) were detected in 8 of the 9 comparison groups (8/9), whereas glycolysis/gluconeogenesis (ko00010) and the pentose phosphate pathway (ko00030) appeared in 7 and 6 comparison groups, respectively. In light of the differential metabolite results described above, these pathways correspond well to the recurrent changes in key sugar metabolites such as D-xylose, D-arabinose, glucose, and D-fructose, suggesting that central carbon metabolism and pentose-related metabolism may contribute to differences in polysaccharide accumulation among materials by supplying carbon sources and precursor substances required for polysaccharide biosynthesis.

Notably, ABC transporters (ko02010) were also repeatedly detected in all nine comparison groups, suggesting that transmembrane transport and intracellular allocation of sugars or related intermediates may participate in tissue- and cultivar-dependent differences in polysaccharide accumulation. By contrast, ascorbate and aldarate metabolism (ko00053), inositol phosphate metabolism (ko00562), and phosphatidylinositol signaling system (ko04070) appeared in 5/9, 4/9, and 4/9 comparison groups, respectively. This suggests that uronic acid conversion, inositol-related metabolism, and its associated signaling processes may contribute to sugar metabolic regulation in some materials, but their recurrence frequency and direct relevance to polysaccharide accumulation were lower than those of the core carbohydrate metabolism pathways described above.

In addition, although metabolic pathways (ko01100) and biosynthesis of secondary metabolites (ko01110) were detected in all comparison groups (9/9), their broad functional scope and limited mechanistic specificity led us to treat them only as background evidence for widespread metabolic reprogramming rather than as core mechanistic pathways explaining differential polysaccharide accumulation.

Taken together, Table 2, Supplementary Figure S2, and Supplementary Table S6A,B show that transcriptome-based KEGG signals generally involved more pathway-mapped DEGs than metabolome-based signals involved DAMs. For the representative recurrent shared pathways listed in Table 2, the numbers of pathway-mapped DEGs ranged from 45 to 312 across comparisons, whereas the numbers of pathway-mapped DAMs were relatively small, ranging from 1 to 4. The adjusted *p* values varied among comparisons and between DEG- and DAM-based enrichment analyses, indicating that some pathways were strongly enriched in specific comparisons whereas others were retained as recurrent shared pathways because both DEGs and DAMs were mapped to them. This pattern is consistent with the observation described in Section 2.3, namely, limited changes in key sugar metabolites accompanied by extensive DEG reprogramming.

Overall, recurrent shared KEGG pathways were mainly concentrated in carbohydrate metabolism, including sugar source utilization, precursor sugar conversion, nucleotide-sugar metabolism, and transport-related processes. These observations provide pathway-level evidence for the involvement of carbohydrate metabolic reprogramming in the observed differences in polysaccharide accumulation. This finding is consistent with the phenotypic result described in Section 2.1, namely, that stem polysaccharide content was significantly higher than that in leaves and that cultivar differences were mainly manifested in stem tissues.

### 2.5. Gene–Metabolite Association Analysis Reveals a Coordinated Pattern Between Transcriptional Changes and Sugar Metabolism Alterations

**Figure 4:**
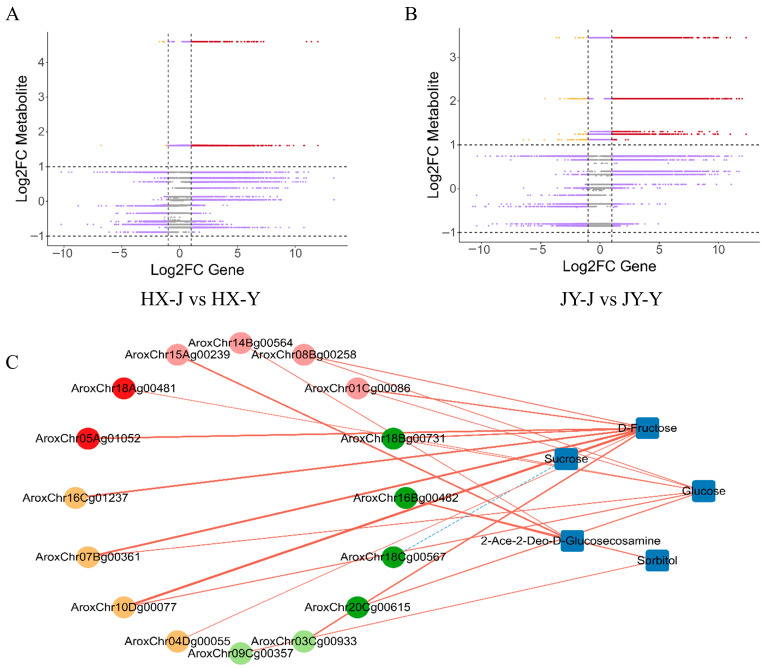
Coordinated variation patterns and key correlation network of differential genes and metabolites in representative tissue comparison groups of *A. roxburghii*. (**A**) Nine-quadrant plot for HX-J vs. HX-Y; (**B**) nine-quadrant plot for JY-J vs. JY-Y. Each point represents a significantly correlated DEG–DAM pair (|r| > 0.8, *p* < 0.05). The x-axis indicates gene log_2_FC, and the y-axis indicates metabolite log_2_FC. Dashed lines indicate the thresholds for differential gene expression (|log_2_FC| = 1) and differential metabolite accumulation (|log_2_FC| = 1). The upper-right and lower-left regions represent coordinated upregulation and coordinated downregulation of genes and metabolites, respectively, whereas the upper-left and lower-right regions indicate opposite directional changes. (**C**) Pearson correlation network of key DEG–DAM pairs. Only significant correlations satisfying |Pearson correlation coefficient| ≥ 0.85 and *p* < 0.01 between candidate genes and candidate metabolites are shown. Circular nodes represent genes, square nodes represent metabolites; red solid lines indicate positive correlations, and blue dashed lines indicate negative correlations. Edge width is proportional to the absolute value of the correlation coefficient. Detailed candidate gene–metabolite correlation pairs are provided in Supplementary Table S7; threshold settings and retained pair numbers are summarized in Supplementary Table S8; metabolite-level network summaries are provided in Supplementary Table S9.

To further evaluate the correspondence between changes in gene expression and metabolite accumulation, Pearson correlation analysis was performed using the quantitative data of DEGs and DAMs across all 18 samples. Significant gene–metabolite pairs were screened using the criteria |r| > 0.8 and *p* < 0.05. Based on this analysis, nine-quadrant plots were generated by integrating the log_2_FC changes in genes and metabolites in each comparison group, thereby characterizing coordinated or opposite patterns between transcriptional changes and metabolic alterations (Figure 4A,B; Supplementary Figure S3).

Given that tissue differences were the major factor affecting polysaccharide accumulation, and that the number and composition of sugar-related DAMs differed between stem–leaf comparisons of different varieties, HX-J vs. HX-Y and JY-J vs. JY-Y were selected as representative nine-quadrant plots for presentation. In HX-J vs. HX-Y, the number of significantly changed sugar-related DAMs was relatively small, mainly reflecting tissue differences at a limited number of sugar metabolism nodes. In contrast, JY-J vs. JY-Y contained more sugar-related DAMs, and JY stems showed the highest polysaccharide content and the largest stem-leaf difference in accumulation; therefore, this comparison reflects more pronounced tissue-related changes in sugar metabolism under a high-polysaccharide-accumulation background. The remaining comparisons are shown in Supplementary Figure S3. In HX-J vs. HX-Y, significantly correlated DEG-DAM pairs tended to be distributed mainly in the upper-right quadrant, corresponding to the “gene upregulation-metabolite increase” region (Figure 4A). This result is consistent with the overall direction of change in this comparison, in which sugar-related differential metabolites were mainly increased in stems. Combined with the differential metabolite analysis described above, only a small number of sugar-related metabolites changed significantly in HX-J vs. HX-Y, mainly including increased D-fructose and D-xylose in stems. In JY-J vs. JY-Y, significantly correlated pairs were also concentrated in the upper-right quadrant (Figure 4B), consistent with the increase in multiple sugar-related metabolites, such as glucose and D-fructose, in stems in this comparison. This suggests a more evident coordinated transcriptional-metabolic change between stems and leaves in the JY variety. Overall, significantly correlated DEG-DAM pairs in each comparison were not randomly distributed but showed a tendency to cluster in quadrants representing coordinated changes, with the “gene upregulation-metabolite increase” region being the most prominent. Meanwhile, the range of gene log_2_FC values was generally larger than that of metabolite log_2_FC values, further indicating that metabolic differences were mainly concentrated at a few key sugar metabolism nodes, accompanied by broader transcriptional reprogramming.

To further focus on key metabolites closely associated with differences in polysaccharide accumulation and their related genes, a core correlation network was constructed around five candidate metabolites and 16 candidate genes based on Pearson correlation analysis between candidate genes and candidate metabolites (Figure 4C). Under the more stringent screening criteria, a compact DEG–DAM correlation network was obtained, and the retained associations were dominated by positive correlations (Supplementary Tables S7 and S8).

D-fructose and glucose were the most highly connected metabolites in this network, sharing several associated genes and forming the main hexose-related association module. Detailed correlation coefficients and gene–metabolite pairs are provided in Supplementary Tables S7–S9. In addition to these two major hexose nodes, 2-acetamido-2-deoxy-D-glucopyranose retained three significant positive correlation edges, connected to AroxChr14Bg00564, AroxChr15Ag00239, and AroxChr16Bg00482, respectively. D-sorbitol retained two significant positive correlation edges, connected to AroxChr09Cg00357 and AroxChr16Bg00482, respectively. By contrast, sucrose retained only one significant correlation edge under the stringent screening criteria and showed a negative correlation with AroxChr18Cg00567 (r = −0.8583), indicating that the candidate network after stringent filtering was dominated by positive correlations (Figure 4C; Supplementary Tables S7 and S9).

Notably, AroxChr01Cg00086, AroxChr07Bg00361, AroxChr08Bg00258, AroxChr10Dg00077, and AroxChr18Bg00731 were significantly correlated with both D-fructose and glucose, indicating that these two major hexose nodes shared a subset of associated genes in the candidate network. Accordingly, the connectivity of D-fructose and glucose among the candidate significant correlation pairs was markedly higher than that of sucrose, D-sorbitol, and 2-acetamido-2-deoxy-D-glucopyranose (Supplementary Table S9). These results identified D-fructose and glucose as highly connected metabolites in the DEG–DAM association network. At the gene level, AroxChr16Bg00482 was significantly correlated with both D-sorbitol and 2-acetamido-2-deoxy-D-glucopyranose, whereas AroxChr18Cg00567 maintained a significant negative correlation only with sucrose (Supplementary Table S7), indicating that, in addition to the main hexose module, there may be a secondary correlation branch composed of sugar alcohol- and amino sugar-related metabolites.

Based on the key nodes retained in Figure 4C, a Pearson correlation heatmap was further generated (Supplementary Figure S4) to illustrate the overall correlation patterns between candidate genes and key metabolites. The heatmap results were generally consistent with the structural features revealed by the correlation network: D-fructose and glucose showed highly similar correlation profiles, and most genes significantly positively correlated with these two metabolites in the network also exhibited consistent positive correlations in the heatmap, whereas their correlations with sucrose were generally weaker or negative. In addition, AroxChr09Cg00357, AroxChr14Bg00564, AroxChr15Ag00239, and AroxChr16Bg00482 showed relatively stronger positive correlations with D-sorbitol and 2-acetamido-2-deoxy-D-glucopyranose, but weaker or opposite correlations with D-fructose and glucose, suggesting that they may represent another correlation pattern distinct from the main hexose module.

Taken together, the nine-quadrant plots, core correlation network, and Pearson correlation heatmap indicate that differences in polysaccharide accumulation among different varieties and tissues of *A*. *roxburghii* are closely associated with coordinated changes between sugar metabolism-related genes and key metabolites. Overall, DEG-DAM pairs showed coordinated change patterns across multiple comparison groups, with D-fructose and glucose forming the main association hubs, D-sorbitol and 2-acetamido-2-deoxy-D-glucopyranose forming a secondary association branch, and sucrose exhibiting a relatively specific correlation pattern dominated by negative associations.

### 2.6. Multivariate Integration by CCA and O2PLS Further Supports Coordinated Relationships Between Transcriptional and Sugar Metabolic Changes

**Figure 5:**
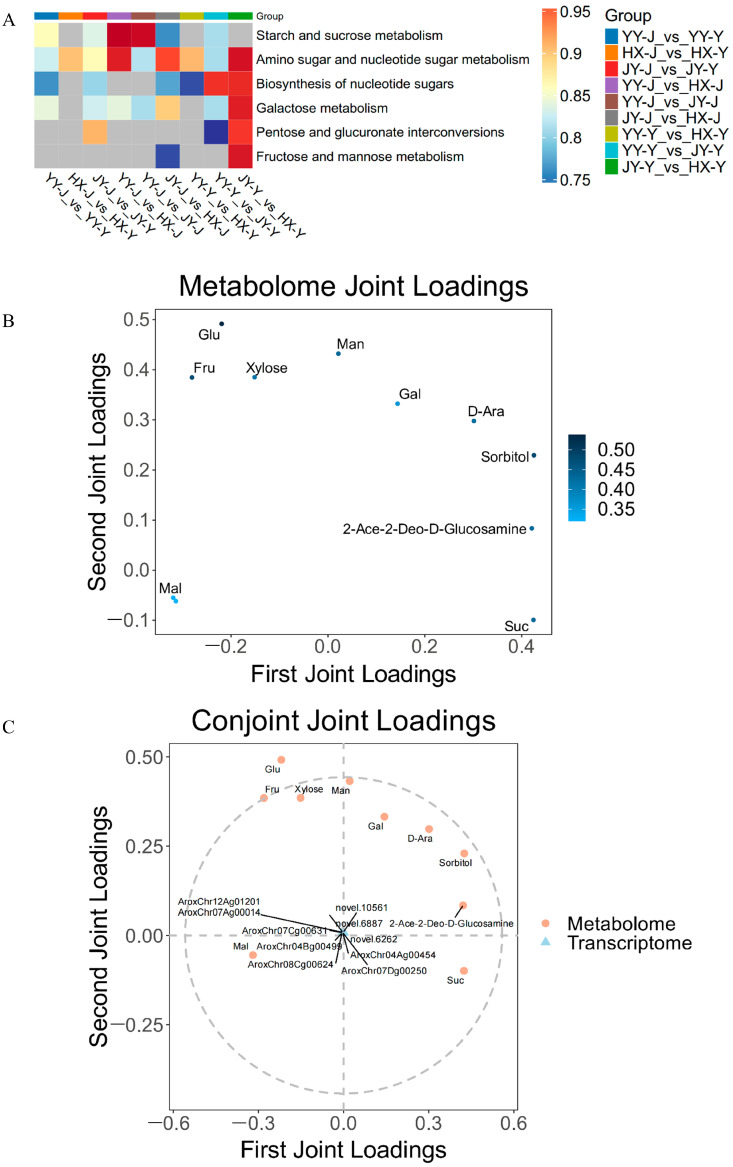
Multivariate transcriptome–metabolome integration analysis associated with polysaccharide accumulation in different cultivars and tissues of *A. roxburghii*. (**A**) Heatmap of the correlations of the first pair of canonical variables (U1V1) obtained by canonical correlation analysis (CCA) for representative carbohydrate metabolism pathways. Color intensity indicates the magnitude of the canonical correlation coefficients between transcriptomic and metabolomic variables; corresponding data are provided in Supplementary Table S10. (**B**) O2PLS metabolomic joint loadings plot. Each point represents one metabolite; the farther a point is from the origin, the greater its contribution to shared transcriptome–metabolome variation. Metabolite details are given in Supplementary Table S11. (**C**) O2PLS joint loadings plot, in which metabolites and transcripts are projected into the same joint space. Different symbols represent metabolites and transcripts, and proximity in position indicates similar contribution patterns to shared variation. The separate transcript joint loadings are shown in Supplementary Figure S5, and transcript details are provided in Supplementary Table S12. Only representative high-loading variables are shown.

To further evaluate the overall correspondence between the transcriptome and sugar metabolome at the multivariate integration level, and to examine whether the gene–metabolite association patterns described above could be consistently supported in multivariate space, canonical correlation analysis (CCA) was performed for shared KEGG pathways in different comparison groups. In addition, O2PLS integration analysis was conducted based on the standardized matrices of all DEGs and DAMs to identify candidate transcripts and metabolites contributing strongly to the shared variation between the two omics datasets (Figure 5; Supplementary Tables S10–S12). It should be noted that, given the limited sample size and the high dimensionality of the omics data, the CCA and O2PLS results were interpreted primarily as exploratory multivariate evidence to assist in explaining coordinated patterns between transcriptional changes and sugar metabolic changes, rather than as direct evidence of causal regulation.

CCA results showed that multiple sugar metabolism-related pathways and transport processes exhibited high correlations for the first pair of canonical variables (U1V1) across several comparison groups (Figure 5A; Supplementary Table S10). These repeatedly included starch and sucrose metabolism (ko00500), amino sugar and nucleotide sugar metabolism (ko00520), biosynthesis of nucleotide sugars (ko01250), fructose and mannose metabolism (ko00051), galactose metabolism (ko00052), and pentose and glucuronate interconversions (ko00040). Overall, the CCA results indicated apparent pathway-level covariation between transcriptional and metabolic changes, particularly in carbohydrate metabolism and transport-related processes (Figure 5A; Supplementary Table S10).

O2PLS analysis further showed clear differences in the distribution of sugar metabolites in the joint loading space, with some metabolites positioned relatively far from the origin, indicating greater contributions to the shared variation between transcriptomic and metabolomic datasets (Figure 5B; Supplementary Table S11). Several sugar-related metabolites, including glucose, D-fructose, sucrose, D-sorbitol, D-mannose, D-arabinose, and D-xylose, contributed substantially to the shared transcriptome–metabolome variation (Figure 5B; Supplementary Table S11). In terms of joint loading distribution, glucose, D-fructose, and D-xylose were mainly located in the negative region of the first joint component, whereas sucrose, D-sorbitol, and 2-acetamido-2-deoxy-D-glucopyranose were mainly located in the positive region of the first joint component. D-mannose, D-galactose, and D-arabinose were positioned in the positive region of the second joint component and displayed relatively high loadings. By contrast, maltose had a negative loading on the first joint component but a relatively small loading on the second component, positioning it closer to the origin overall and indicating a weaker contribution to shared variation than the higher-loading metabolites described above (Figure 5B; Supplementary Table S11).

In contrast, the joint loadings of transcriptomic variables were more concentrated overall, with only a few transcripts showing relatively high loadings (Supplementary Figure S5; Supplementary Table S12). Representative high-loading transcripts included novel.6887, AroxChr07Ag00014, AroxChr12Ag01201, AroxChr07Cg00631, AroxChr07Dg00250, AroxChr04Ag00454, and AroxChr08Cg00624. Among these candidate transcripts, AroxChr07Ag00014 was associated with ko00500, AroxChr03Dg00597 and AroxChr03Ag00615 were associated with ko00052, and AroxChr01Ag01443 was associated with ko02010.

In the conjoint joint loadings plot, metabolites and transcripts were projected into the same joint space (Figure 5C). Glucose, D-fructose, and D-xylose were mainly distributed in the upper-left region, whereas sucrose, D-sorbitol, and 2-acetamido-2-deoxy-D-glucopyranose were mainly located on the right side. D-mannose, D-galactose, and D-arabinose were mainly distributed in the upper region. By contrast, transcript variables were more concentrated near the center of the plot, with only a few high-loading transcripts being labeled. Overall, the conjoint joint loadings plot further showed that metabolomic variables were more widely dispersed in the joint space, whereas transcriptomic variables displayed a more concentrated distribution (Figure 5C).

Taken together, the CCA and O2PLS results indicate that differences in polysaccharide accumulation among cultivars and tissues of *A. roxburghii* exhibit relatively consistent transcript–metabolite covariation at both the pathway level and the shared-variation level. In particular, starch and sucrose metabolism, amino sugar and nucleotide sugar metabolism, biosynthesis of nucleotide sugars, fructose and mannose metabolism, galactose metabolism, pentose and glucuronate interconversions, and related transport processes repeatedly showed high canonical correlation coefficients across multiple comparison groups, whereas metabolites such as glucose, D-fructose, sucrose, D-sorbitol, D-mannose, D-galactose, and D-xylose contributed substantially to the shared variation.

### 2.7. WGCNA Identifies Co-Expression Modules Associated with Sugar-Related Traits

**Figure 6:**
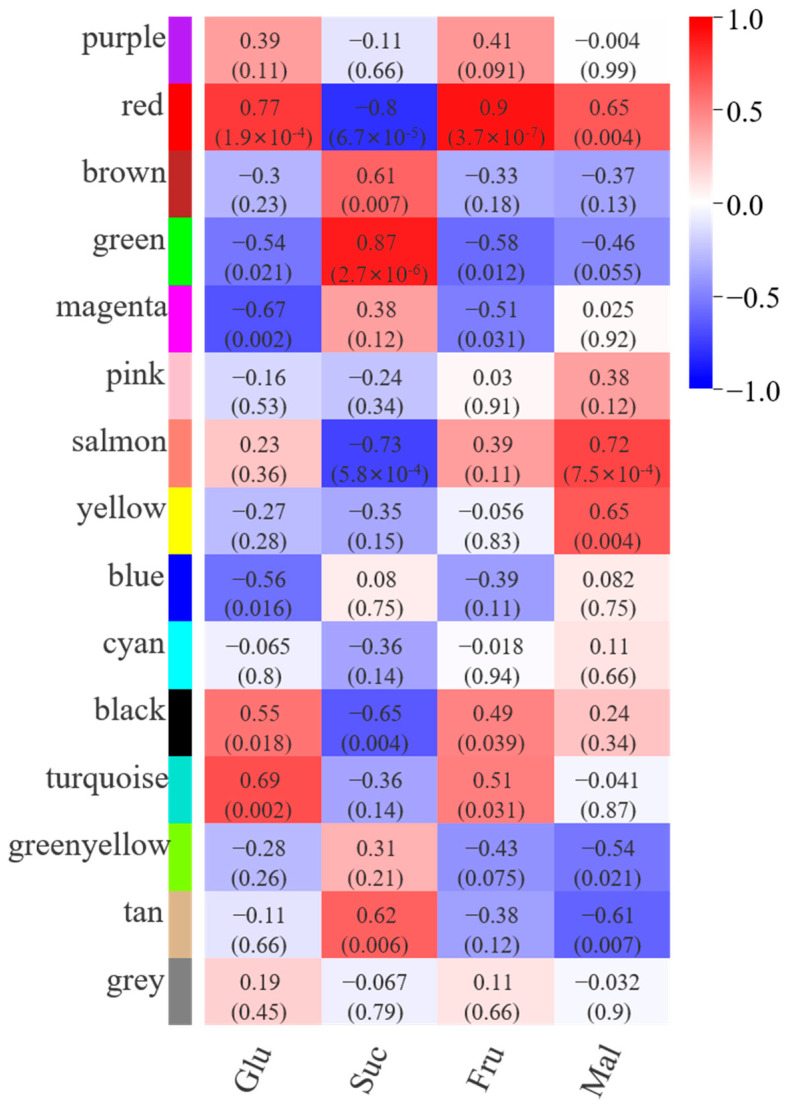
Correlation analysis between WGCNA co-expression modules and representative sugar metabolite contents in *A. roxburghii*. Colors indicate Pearson correlation coefficients, with red representing positive correlations and blue representing negative correlations; color intensity is proportional to the absolute value of the correlation coefficient. Values within each cell indicate the correlation coefficient, and values in parentheses indicate significance levels.

To systematically dissect gene co-expression regulatory units closely associated with sugar metabolic reprogramming and polysaccharide accumulation in *A. roxburghii*, WGCNA was performed based on the complete transcriptomic dataset. Multiple co-expression modules with distinct expression patterns were identified (Supplementary Figure S7). Pearson correlation analysis between module eigengenes and the contents of glucose (Glu), fructose (Fru), maltose (Mal), and sucrose (Suc) revealed that the red, green, salmon, and tan modules were significantly associated with sugar metabolism. (Figure 6).

To resolve the internal regulatory architecture of these modules, intramodular connectivity (k_Within_) was calculated for all genes. Core hub genes were subsequently screened using a multi-parameter filtering strategy: module membership (MM > 0.8), gene significance (|GS| > 0.6), and top 10 ranking by k_Within_. A total of 10 hub genes were identified in each of the four modules, and their functional annotations are summarized in Supplementary Table S13. The MM-GS scatter plots for each module are provided in Supplementary Figures S8–S11.

Among these modules, the red module showed the closest and most specific association with sugar metabolism. It displayed significant positive correlations with both glucose and fructose and a significant negative correlation with sucrose; for all three traits, both the strength and significance of the correlations ranked first among all modules. In addition, this module also showed a significant positive correlation with maltose, with the second-highest correlation strength among all modules. Functional integration of hub genes within the red module revealed an interconnected regulatory network driving carbon flux, matrix assembly, and proteostasis (Supplementary Table S13). Specifically, *KA120* (AroxChr06Cg00433), encoding a PPi-dependent phosphofructokinase (EC:2.7.1.90), is a candidate gene potentially involved in glycolytic carbon flux regulation. Concurrently, LUH (AroxChr12Cg00253), a WD40 repeat and LUFS domain transcriptional corepressor, points to modulated cell wall pectin methylesterification and polysaccharide matrix assembly. This carbon allocation framework is reinforced by protein quality control and turnover machinery, including the proteasome subunit PBA1 (AroxChr06Bg00106), the ufmylation ligase UFL1 (AroxChr01Ag00667), and a RING-type E3 ligase (AroxChr14Cg00359), which collectively maintain metabolic enzyme stability during rapid sugar turnover. In addition, hub components mediating pre-mRNA processing (COILIN, AroxChr12Ag00661), translation (RPS5B, AroxChr05Bg00147), nucleocytoplasmic transport (importin-11-like CaLB protein, AroxChr12Dg00433), and lipid export (LTPG2, AroxChr20Dg00497) support basal cellular integrity, indicating that the red module is associated with hexose enrichment and polysaccharide synthesis through synchronized metabolic and post-translational coordination.

In sharp contrast to the regulatory pattern of the red module, the green module showed a highly significant positive correlation with sucrose and significant negative correlations with glucose and fructose, making it a representative module of sucrose accumulation. Hub genes in this module converged on maintaining source-tissue energetics, organelle proteostasis, and tissue development (Supplementary Table S13). Key metabolic and structural factors included plastid-localized regulators such as PDS3 (AroxChr16Cg00064, phytoene desaturase) and ACA1 (AroxChr05Cg00453, chloroplastic alpha carbonic anhydrase), alongside organellar proteostasis factors including J2 (AroxChr07Cg00268, DnaJ co-chaperonin) and NFS1 (AroxChr12Cg01228, Fe-S cluster assembly). Transcriptional and developmental control was represented by HB-1 (AroxChr19Cg00242, HD-Zip I TF) and CYCT1 (AroxChr04Bg00981, cyclin T). Furthermore, mitochondrial channel protein VDAC1 (AroxChr09Dg00554) and cellular growth regulator TIP1 (AroxChr06Cg00625) were co-expressed, suggesting that the green module sustains sucrose retention and export in source organs by preserving plastid function, mitochondrial metabolite exchange, and organelle protein homeostasis.

For maltose metabolism, the salmon and tan modules exhibited completely opposite regulatory patterns: the salmon module showed the strongest significant positive correlation with maltose, whereas the tan module showed the strongest significant negative correlation with maltose. The salmon module integrated active protein synthesis, energy charging, and cell wall matrix modification to support maltose accumulation. Its core network encompassed translational components (US3X, US19Y, novel.7053, and mitochondrial L45), adenylate kinase ADK1 (AroxChr16Bg00174) for energy transfer, ubiquitin conjugating enzyme UBC10 (AroxChr16Cg00095), and critically, a homogalacturonan methyltransferase (AroxChr12Ag00295) directly involved in pectin backbone methylation and structural polysaccharide assembly. Conversely, the tan module was dominated by photosynthetic light reactions and plastid protein synthesis, led by ferredoxin FED A (AroxChr12Ag00767), plastid ribosomal subunit EMB3126 (AroxChr01Bg01286), PPR protein PGR3 (AroxChr17Dg00213), and stromal protease adapter DUF760-4 (AroxChr16Bg00774), complemented by photomorphogenic factors (COP9, CCD1), iron transport (YSL3), and cytoskeletal elements (tubulin alpha-6). The negative correlation of the tan module with maltose indicates that high photosynthetic capacity in source tissues favors sucrose synthesis and primary carbon export over maltose storage.

Overall, WGCNA revealed a multi-tiered transcriptional coordination network driving carbohydrate metabolism in *A. roxburghii*. The red module governs hexose phosphorylation and cell wall precursor supply, the green module maintains sucrose homeostasis, and the opposing salmon and tan modules fine-tune maltose partitioning and pectin modification.

### 2.8. An Integrated Working Model for Polysaccharide Accumulation Among Different Cultivars and Tissues of A. roxburghii

**Figure 7:**
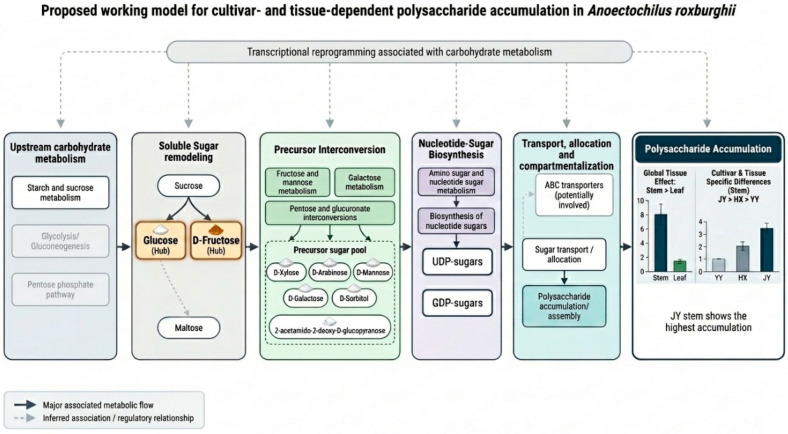
Proposed working model for cultivar- and tissue-dependent polysaccharide accumulation in *A. roxburghii.* The model integrates the major findings from polysaccharide quantification, transcriptomics, targeted sugar metabolomics, correlation analysis, CCA, O2PLS, and WGCNA. It summarizes the main processes associated with differential polysaccharide accumulation, including upstream carbohydrate metabolism, soluble sugar remodeling, precursor interconversion, nucleotide-sugar biosynthesis, and transport/allocation-related processes. Among the soluble sugar nodes, glucose and D-fructose are highlighted as major hub metabolites based on their recurrent differential accumulation and prominent contribution in correlation and multivariate integration analyses. Overall, the integrated pattern is associated with higher polysaccharide accumulation in stems than in leaves, with cultivar differences being more evident in stems and the highest accumulation observed in JY stem. This figure was created with the assistance of Gemini Pro Image and finalized by the authors.

To integrate the transcriptomic, sugar metabolomic, and multi-omics results described above, a working model for polysaccharide accumulation among different cultivars and tissues of *A. roxburghii* was constructed (Figure 7). Taken together, the results from Section 2.1, Section 2.2, Section 2.3, Section 2.4, Section 2.5, Section 2.6 and Section 2.7 show that polysaccharide accumulation displayed marked tissue dependence and cultivar dependence, with stems generally containing higher polysaccharide levels than leaves and cultivar differences being mainly manifested in stems; among all materials, JY stems showed the highest level of polysaccharide accumulation. The integrated multi-omics results consistently indicated that these differences were mainly associated with transcriptional reprogramming and sugar metabolic remodeling related to carbohydrate metabolism.

Specifically, Figure 7 summarizes an integrated framework composed of upstream carbon metabolism, soluble sugar remodeling, precursor sugar interconversion, nucleotide sugar donor formation, and transport/allocation processes. Within this framework, starch and sucrose metabolism emerged as the most stably associated core pathway, whereas glycolysis/gluconeogenesis and the pentose phosphate pathway represented upstream supporting processes related to carbon flux partitioning. During sucrose turnover and soluble sugar remodeling, glucose and D-fructose showed prominent performance in terms of recurrence frequency among differential metabolites, network connectivity, and O2PLS contribution, indicating that they function as key hub metabolites linking transcriptional changes to differences in sugar metabolism. Meanwhile, fructose and mannose metabolism, galactose metabolism, pentose and glucuronate interconversions, and amino sugar and nucleotide sugar metabolism were jointly associated with remodeling of the precursor sugar pool; nucleotide sugar biosynthesis further suggested that the supply of UDP-/GDP-sugars may participate in downstream polysaccharide accumulation processes. Together with transport-, allocation-, and compartmentalization-related signals, Figure 7 indicates that differential polysaccharide accumulation among cultivars and tissues of *A. roxburghii* is closely associated with multi-layered carbohydrate metabolic reprogramming.

## 3. Discussion

Polysaccharides are major quality-related bioactive constituents of *A. roxburghii* and are closely associated with pharmacological activities such as hypoglycemic, antioxidant, immunomodulatory, and hepatoprotective effects [1,2,3]. The present study provides a multi-omics view of how carbohydrate metabolism contributes to tissue- and cultivar-dependent polysaccharide accumulation. Rather than reflecting the accumulation of a single sugar metabolite, the observed variation appears to result from coordinated changes in carbon allocation, sucrose turnover, precursor sugar interconversion, nucleotide-sugar formation, and sugar transport/allocation. These findings extend previous studies that mainly focused on extraction, purification, structural characterization, and bioactivity evaluation of *A. roxburghii* polysaccharides [2,3,5], and provide a metabolic framework for understanding quality formation in this medicinal plant. Across medicinal Orchidaceae plants, conserved carbohydrate modules appear to underlie polysaccharide accumulation, but the organ context and regulatory emphasis differ among species.

The preferential accumulation of crude polysaccharides in stems can be interpreted from the perspective of source–sink carbon allocation. Leaves are primarily responsible for photosynthetic carbon assimilation and sucrose export, whereas stems may act as sink-related tissues where imported sugars are unloaded, converted, and incorporated into storage or structural carbohydrates. Therefore, higher polysaccharide accumulation in stems likely reflects stronger sink activity and more active conversion of transport sugars into polysaccharide precursors, rather than simple retention of soluble sucrose. Similar tissue-biased polysaccharide accumulation has been reported in Dendrobium moniliforme, in which stems showed the highest polysaccharide content, and sugar metabolism-related genes such as Susy and SPS displayed tissue-specific expression patterns [7]. These observations suggest that carbon sink strength, sugar unloading, and carbohydrate conversion capacity are important determinants of stem-biased polysaccharide accumulation in *A. roxburghii* and other medicinal orchids.

The tissue-dependent expression of cultivar differences suggests that genetic background may influence polysaccharide accumulation mainly by modulating sink strength and carbon conversion capacity. Leaf carbohydrate metabolism is constrained by its fundamental role in photosynthesis and assimilate export, and may therefore remain relatively conserved among cultivars. In contrast, stems may show greater plasticity in sugar unloading, interconversion, storage, and cell-wall carbohydrate deposition. Thus, cultivar-specific carbon partitioning is more likely to be manifested in sink-related tissues. Because the three cultivars are clonally propagated cultivated materials, their metabolic differences may reflect inherited divergence and historical selection for growth, morphology, biomass, or medicinal quality.

Comparable evidence from medicinal orchids and other medicinal plants further supports the importance of organ context and carbohydrate metabolic reprogramming in polysaccharide-related quality formation. During taproot thickening in *Panax notoginseng*, starch and sucrose metabolism together with cell-wall metabolism were identified as important processes associated with storage organ development [8]. In *Dendrobium nobile*, transcriptomic and metabolomic analyses also revealed a close relationship between carbohydrate metabolism and the formation of medicinal components [9]. Similar organ- and material-specific accumulation patterns have also been reported in *Polygonatum*, where integrated omics analyses revealed clear differences in polysaccharides and other bioactive metabolites among organs and developmental stages [10]. Together, these studies indicate that tissue-specific carbon allocation, carbohydrate conversion, and developmental or material background are common factors shaping polysaccharide accumulation in medicinal plants.

The central role of glucose and fructose is biologically reasonable because these hexoses are direct products of sucrose cleavage and occupy key positions in plant carbohydrate metabolism. They can be phosphorylated and further directed into glycolysis, the pentose phosphate pathway, and nucleotide-sugar biosynthesis, thereby linking sucrose utilization with polysaccharide precursor supply. At the molecular level, this transition is directly driven by key hub enzymes such as KA120 (AroxChr06Cg00433), which encodes a PPi-dependent phosphofructokinase (PFP, EC:2.7.1.90). By catalyzing the interconversion of fructose 6-phosphate and fructose 1,6-bisphosphate, KA120 functions as a crucial metabolic valve that channels glycolytic carbon flux and hexose phosphorylation directly toward nucleotide-sugar precursor pools. In this sense, high polysaccharide accumulation depends more on rapid sucrose turnover and hexose utilization driven by enzymes like KA120 than on sucrose retention itself. This interpretation is indirectly supported by previous studies in *A. roxburghii*, in which glucose-containing polysaccharide fractions were isolated and shown to possess significant biological activities [2].

Besides hexoses, the involvement of pentose-, mannose-, galactose-, amino sugar-, and nucleotide-sugar-related metabolism suggests that polysaccharide accumulation in *A. roxburghii* is accompanied by remodeling of the precursor sugar pool. Plant polysaccharide assembly requires multiple monosaccharide precursors and activated sugar donors, such as UDP- and GDP-sugars, which serve as substrates for glycosyltransferase-mediated polymer formation. Therefore, enhanced precursor interconversion and nucleotide-sugar production may provide the biochemical basis for both increased polysaccharide accumulation and potential variation in polysaccharide composition. This point is particularly relevant because *A. roxburghii* polysaccharides exhibit pronounced structural heterogeneity, with different fractions varying in molecular weight, monosaccharide composition, glycosidic linkage type, and chain conformation [2,3,5]. Similar observations have been reported in *Polygonatum* species, where genes and pathways involved in sugar precursor interconversion and nucleotide-sugar metabolism were associated with polysaccharide accumulation [11,12]. Together, these findings suggest that precursor sugar interconversion and activated sugar donor formation are common metabolic bases for differential polysaccharide accumulation in medicinal plants.

An important implication of the integrated analysis is that polysaccharide accumulation appears to be a network-regulated trait. The relatively limited variation in free sugar metabolites suggests that metabolic output is concentrated at several key carbohydrate nodes. However, these metabolic nodes are embedded in broad transcriptional changes involving sugar conversion, transport, nucleotide-sugar formation, and cell-wall-related processes. Thus, cultivar- and tissue-dependent polysaccharide accumulation in *A. roxburghii* is unlikely to be controlled by a single metabolite or pathway; rather, it represents the integrated output of coordinated carbon metabolic regulation. Similar multi-layered regulatory patterns have been reported in *Cynomorium songaricum*, *Polygonatum odoratum, and Panax ginseng* [13,14,15], supporting the view that medicinal polysaccharide accumulation is generally controlled by coordinated metabolic networks.

The co-expression network analysis further provided candidate transcriptional modules potentially linking sugar partitioning with polysaccharide accumulation. Specifically, the red module, which exhibited the strongest positive correlation with hexose accumulation and polysaccharide content, is organized around a dual metabolic-transcriptional engine. Beyond the glycolytic driver KA120 (PFP), this module features LUH (AroxChr12Cg00253), a WD40 repeat and LUFS domain transcriptional corepressor known to modulate cell wall pectin methylesterification and polysaccharide matrix assembly. This primary carbon allocation framework is critically supported by an intrinsic protein quality control network, including the proteasome alpha subunit PBA1 (AroxChr06Bg00106), the ufmylation ligase UFL1 (AroxChr01Ag00667), and a RING-type E3 ligase (AroxChr14Cg00359). These factors likely protect essential carbohydrate metabolic enzymes from degradation and misfolding during intensive sugar turnover in sink tissues. Furthermore, the salmon module directly links energetic charging with matrix synthesis through ADK1 (AroxChr16Bg00174, adenylate kinase) and a homogalacturonan methyltransferase (AroxChr12Ag00295), which directly catalyzes pectin backbone methylation and structural polysaccharide deposition. In sharp contrast, the green and tan modules are dominated by plastid-localized energetics (e.g., PDS3, ACA1) and photosynthetic light reactions (e.g., ferredoxin FED A, PGR3), reflecting a source-tissue status that preserves sucrose for long-distance transport rather than stem polysaccharide deposition. This is consistent with studies in polyploid *Dendrobium* showing that sugar transport and cell-wall polysaccharide biosynthesis are important components of high-polysaccharide phenotypes [16]. Together, these specific hub gene networks demonstrate that sugar partitioning, precursor utilization, cell-wall pectin modification, and organelle proteostasis systematically determine tissue- and cultivar-dependent polysaccharide accumulation in *A. roxburghii*.

To place our findings in a broader context of medicinal Orchidaceae plants, we further compared the present results with published multi-omics studies related to carbohydrate metabolism and polysaccharide accumulation. In *Bletilla striata*, integrated multi-omics analysis showed that suitable light intensity regulates polysaccharide biosynthesis through carbon metabolism in leaves, indicating the importance of photosynthetic carbon assimilation and downstream carbohydrate metabolism in orchid polysaccharide accumulation [17]. Together with previous studies in medicinal orchids, these findings suggest that carbon fixation, starch and sucrose metabolism, sugar interconversion, nucleotide-sugar formation, glycosyltransferase-mediated polymer assembly, and sugar transport may represent conserved carbohydrate metabolic modules associated with polysaccharide accumulation. However, the organ context in which these modules operate differs among species. *Dendrobium* studies have mainly emphasized stem-associated polysaccharide accumulation and carbohydrate conversion, whereas *Bletilla striata* highlights light-regulated carbon metabolism in leaves. In contrast, *A. roxburghii* is used medicinally as a whole herb, and both stems and leaves contribute to its medicinal quality. Therefore, the stem–leaf source–sink relationship is particularly important in this species. The distinct polysaccharide accumulation patterns observed among the cultivars examined in this study further suggest that genotype-dependent carbon partitioning is a key factor underlying tissue-specific polysaccharide accumulation in *A. roxburghii*. Thus, the species-specific feature of *A. roxburghii* lies not in the presence of completely unique carbohydrate pathways, but in the stem-dominant and cultivar-amplified deployment of conserved carbohydrate metabolic modules under a whole-herb medicinal context.

Recent studies in *A. roxburghii* further support the idea that central carbon metabolism in this species is highly plastic. A recent salt-stress study in *A. roxburghii* reported extensive reprogramming of central carbon metabolism and soluble-sugar accumulation, although it did not directly quantify polysaccharide accumulation [18]. Integrated transcriptomic and metabolomic analysis of high-temperature-acclimated *A. roxburghii* revealed coordinated changes in carbon fixation, amino acid metabolism, lipid metabolism, carbohydrate metabolism, and flavone/phenylpropanoid biosynthesis [19], whereas transcriptomic profiling under the combined experimental context of drought stress and exogenous spermidine application identified differential expression associated with phenylpropanoid biosynthesis, starch and sucrose metabolism, and plant hormone signal transduction [20]. Sugar-free tissue culture was associated with changes in photosynthesis-related gene expression and specialized metabolite profiles [21]. Separately, transcriptomic profiling during in vitro flowering identified stage-dependent expression changes enriched in metabolic, starch and sucrose metabolism, secondary-metabolite biosynthesis, DNA replication, and hormone-signaling pathways [22]. These findings indicate that carbon metabolism in *A. roxburghii* can be reshaped by genotype, tissue type, developmental status, and environmental cues. Moreover, *A. roxburghii* polysaccharides have been reported to possess anti-inflammatory, immunomodulatory, hepatoprotective, and gut microbiota-regulatory activities [4]. Thus, elucidating the molecular basis of polysaccharide accumulation in different cultivars and tissues is important not only for understanding carbohydrate metabolic regulation in this species but also for quality evaluation and medicinal utilization. These comparisons further indicate that our study extends previous orchid multi-omics research by revealing cultivar- and tissue-specific regulation of polysaccharide accumulation in *A. roxburghii*, highlighting its species-specific value as a photosynthetic whole-herb medicinal orchid.

It should be emphasized that the present study determined the content of ethanol-precipitated water-extracted crude polysaccharides, and the results mainly reflect the overall level of crude polysaccharide accumulation among cultivars and tissues rather than the absolute content of specific purified polysaccharide fractions. At the same time, the sugar metabolomic analysis mainly detected free sugars and related small molecules, which are more suitable for interpreting carbon flux partitioning and precursor supply status, but cannot be directly equated with the monosaccharide composition and fine structure of the final polysaccharide polymers. In addition, *A. roxburghii* polysaccharides themselves exhibit substantial structural heterogeneity, and different fractions may differ significantly in molecular weight, monosaccharide composition, glycosidic linkage type, and chain conformation [2,3,5]. Therefore, the crude polysaccharide changes observed here are better interpreted in terms of overall accumulation trends rather than the behavior of any single structurally defined polysaccharide species. At present, functional interpretations of candidate genes and modules are still mainly based on annotation and correlation analyses, and further confirmation will require enzyme activity assays, structural characterization of purified fractions, and genetic functional validation. In the future, it will be necessary to combine targeted enzyme activity measurements, fine structural characterization of polysaccharides, and functional verification of candidate genes to establish more direct links among “precursor supply–polymer assembly–structure formation–bioactivity expression”.

Overall, the present study supports a relatively clear hierarchical explanatory framework: differences in polysaccharide accumulation among cultivars and tissues of *A. roxburghii* are mainly driven by upstream sugar source supply, sucrose turnover and soluble sugar remodeling, precursor sugar interconversion, nucleotide-sugar donor formation, and transmembrane transport and compartmentalized allocation, all accompanied by extensive transcriptional reprogramming. Within this framework, stems represent the key tissues for differential polysaccharide accumulation, and JY is a representative cultivar with strong high-polysaccharide accumulation potential. Together with previous studies on the structural diversity and broad pharmacological activities of *A. roxburghii* polysaccharides [1,2,3], and integrated omics studies in other medicinal plants focusing on tissue-specific accumulation and carbon metabolic regulation [7,8,13], the high-polysaccharide tissues, superior cultivar, and related carbohydrate metabolic modules identified in this study provide a useful basis for subsequent germplasm screening, precision cultivation regulation, and functional product development of high-quality *A. roxburghii*. If further combined with environmental factors or beneficial microbial regulation strategies [23], it may become possible to coordinately improve polysaccharide quality in *A. roxburghii* from multiple dimensions, including genetic background, tissue specificity, and metabolic regulation. Therefore, compared with other medicinal orchids, the species-specific value of *A. roxburghii* lies in its whole-herb medicinal use and in the stem-dominant, cultivar-amplified deployment of conserved carbohydrate metabolic modules.

From an applied perspective, the hub genes and metabolic nodes identified here provide concrete, actionable targets for molecular breeding and quality regulation. The red module hub gene *KA120* (encoding PPi-dependent phosphofructokinase) and the green module sucrose-associated genes represent promising candidates for marker-assisted selection (MAS) of high-polysaccharide germplasm, as their expression directly reflects carbon flux toward polysaccharide precursors. For cultivar improvement, the stem-biased accumulation pattern and the JY-specific metabolic signature—marked by elevated hexoses and enhanced nucleotide-sugar metabolism—offer quantitative indices for early-stage, non-destructive screening of superior lines. Regarding quality regulation, glucose and fructose, together with the core pathways of starch and sucrose metabolism and nucleotide-sugar biosynthesis, establish a metabolic roadmap for targeted intervention. Future precision cultivation may thus be designed to modulate sucrose turnover and precursor supply—via agronomic management, environmental optimization, or genetic manipulation of key hub genes such as *KA120* and *LUH*—to coordinately enhance polysaccharide content in a tissue-specific manner.

## 4. Materials and Methods

### 4.1. Plant Materials and Experimental Design

Tissue-cultured plantlets of three *A. roxburghii* cultivars, Hongxia (HX), Jianye (JY), and Yuanye (YY), were used as experimental materials in this study. All plantlets were purchased from Guangdong Yanguilai Wellness Industry Co., Ltd. (Meizhou, Guangdong, China), originated from the same batch, and were 7–8 months old. After purchase, all materials were acclimated in a laboratory tissue culture room for approximately 10 d under uniform conditions of 28 °C, a 12 h light/12 h dark photoperiod, and a light intensity of 1500 lx.

A two-factor design consisting of three cultivars × two tissues (leaf and stem) was adopted, with three biological replicates per group, yielding a total of 18 samples. Each biological replicate consisted of pooled tissues from the same organ position of three plants with similar growth status. Leaf samples were collected from the first fully expanded mature functional leaf below the apical leaf, whereas stem samples were taken from the upper-middle stem segment while avoiding nodes.

For each cultivar–tissue combination, the same biological replicate pool was used for all downstream analyses. After collection, each pooled biological sample was thoroughly mixed and divided into separate matched aliquots. One aliquot was immediately frozen in liquid nitrogen and stored at −80 °C for RNA extraction and transcriptome sequencing; another aliquot was immediately frozen in liquid nitrogen and stored at −80 °C for targeted sugar metabolomic analysis; and a further aliquot from the same biological replicate pool was used for crude polysaccharide determination after drying. Thus, transcriptomic, metabolomic, and polysaccharide-content data were generated from matched aliquots derived from the same biological replicate pools. All samples were collected within the same time window and processed immediately as described above.

### 4.2. Determination of Crude Polysaccharide Content

Polysaccharide content was determined using the phenol–sulfuric acid method. After oven-drying, samples were ground into powder, and 1.0 g of each sample was extracted with purified water at a solid–liquid ratio of 1:50 at 80 °C for 120 min. After filtration, 2 mL of filtrate was mixed with 10 mL of absolute ethanol and allowed to stand at 4 °C for 24 h to precipitate polysaccharides. The mixture was then centrifuged at 3000× *g* for 15 min, the supernatant was discarded, and the precipitate was redissolved in 30 mL of purified water as the test solution. No defatting, decolorization, or deproteinization steps were performed during the extraction process.

Glucose was used as the standard for generating a calibration curve. After color development using the phenol–sulfuric acid method, absorbance was measured at 490 nm using a BioTek Epoch full-wavelength microplate reader. Three biological replicates were analyzed for each group, and polysaccharide content was expressed on a dry weight basis as mg g^−1^ DW. It should be noted that the values obtained in this study represent the content of ethanol-precipitated water-extracted crude polysaccharides rather than that of purified polysaccharide fractions.

### 4.3. RNA Extraction, Quality Assessment, Library Construction, and Transcriptome Sequencing

Transcriptome sequencing was performed by Metware Biotechnology Co., Ltd. (Wuhan, China). Total RNA was extracted from plant samples using the CTAB method combined with pBIOZOL reagent for plant total RNA extraction. The main reagents included cetyltrimethylammonium bromide (CTAB; A600108-0500, Sangon Biotech, Shanghai, China), pBIOZOL reagent for plant total RNA extraction (BSC55M1, Bioer Technology, Hangzhou, China), RNase-free DNase I (M0303S, New England Biolabs, Ipswich, MA, USA), and DNase I Reaction Buffer (B0303S, New England Biolabs). Genomic DNA contamination was removed by DNase I treatment, and the extracted RNA was dissolved in DEPC-treated water.

RNA concentration was quantified using a Qubit 4.0 fluorometer, and RNA integrity was assessed using a Qsep400 high-throughput nucleic acid/protein analysis system. All RNA samples passed the quality-control criteria for library construction. The RNA concentrations ranged from 20 to 95 ng/μL, the total RNA amounts ranged from 0.94 to 4.75 μg, and the RNA integrity values ranged from 8.4 to 8.6 across the 18 samples. Detailed RNA quality information for each sample is provided in Supplementary Table S14.

mRNA was enriched from total RNA using Oligo(dT) magnetic beads with VAHTS mRNA Capture Beads 2.0 (N403-C4, Vazyme, Nanjing, China). The purified mRNA was fragmented and reverse-transcribed using random hexamer primers to synthesize first-strand cDNA, followed by second-strand cDNA synthesis, end repair, dA-tailing, adapter ligation, PCR amplification, and purification. RNA-seq libraries were constructed using the VAHTS Universal V10 RNA-seq Library Prep Kit for Illumina (Premixed version; NR616-02, Vazyme, Nanjing, China) with MGI-compatible adapters and primers. For each sample, 500 ng of total RNA was used as input. The target insert size was approximately 300 bp, corresponding to a selected insert range of 250–350 bp, and 13 PCR cycles were performed. The libraries were non-strand-specific.

After library quality assessment using Qubit-based quantification and Qsep400 fragment-size analysis, the double-stranded DNA libraries were denatured and circularized to generate single-stranded circular DNA libraries. DNA nanoballs were prepared by phi29 amplification and loaded onto sequencing chips. Sequencing was performed on the DNBSEQ-T7 platform using a paired-end 150 bp sequencing strategy.

### 4.4. Transcriptomic Data Processing and Differential Expression Analysis

Raw sequencing reads were quality-filtered using fastp v0.23.2 [24] to remove adapter sequences and low-quality reads. Paired reads were discarded if either read contained more than 10% ambiguous bases (N), or if the number of low-quality bases (Q ≤ 20) exceeded 50% of the total bases in either read. All downstream analyses were performed using clean reads.

Across the 18 RNA-seq libraries, 38,283,850–58,517,142 raw reads and 37,850,956–57,572,400 clean reads were obtained per sample, corresponding to 5.68–8.64 Gb clean bases. The sequencing error rate was 0.02% for all libraries. The Q20 and Q30 values ranged from 99.13% to 99.42% and from 96.79% to 97.84%, respectively, and the GC content ranged from 45.09% to 46.11%. These results indicated high sequencing quality for all libraries. Detailed sequencing-quality information for each sample is provided in Table S14.

Clean reads were aligned to the chromosome-level autotetraploid reference genome assembly of *A. roxburghii* cultivar ‘Jinkang No. 1’ (GenBank accession: GCA_051820535.1, Assembly: ASM5182053v1; available via NCBI: https://identifiers.org/ncbi/insdc.gca:GCA_051820535.1, accessed on 28 November 2025) [25] using HISAT2 v2.2.1 [26] default parameters. Gene models were defined based on the corresponding genome structural annotation file. The overall mapping rate ranged from 87.82% to 99.06%, and uniquely mapped reads accounted for 56.49–71.29% of total reads. Detailed mapping statistics for each library are provided in Table S14. Given the autotetraploid nature of *A. roxburghii* and the high sequence identity among homologous chromosomes and duplicated gene copies, multi-mapped reads accounted for approximately 30% of the total aligned reads. To prevent expression quantification ambiguity and avoid false-positive differential expression statistics caused by cross-mapping among homologous alleles or paralogs, gene-level expression quantification was performed using featureCounts v2.0.3 [27] by strictly retaining only uniquely mapped reads. Transcript assembly and novel transcript prediction were performed using StringTie v2.1.6 [28] when applicable.

Global expression relationships among samples were evaluated by Pearson correlation analysis and principal component analysis (PCA). Differential expression analysis was performed using DESeq2 v1.22.1 [29,30] based on raw read counts. Differentially expressed genes (DEGs) were identified using the criteria |log_2_FC| ≥ 1 and false discovery rate (FDR) < 0.05. Functional annotation of DEGs was performed against the KEGG, GO, and KOG databases, and enrichment analyses were conducted for KEGG pathways and GO terms. Because this study focused primarily on carbohydrate metabolism-related processes, the main text emphasizes KEGG pathway results related to polysaccharide and sugar metabolism.

### 4.5. Extraction, GC–MS Detection, and Quantification of Sugar Metabolites

Targeted sugar metabolomic analysis was performed by Metware Biotechnology Co., Ltd (Wuhan, China). based on a Gas Chromatography-Mass Spectrometry (GC–MS) platform for the targeted profiling of 32 sugars and sugar-related metabolites. After vacuum freeze-drying, samples were ground at 30 Hz for 1.5 min using a ball mill (MM400, Retsch, Haan, Germany). A 20 mg aliquot of sample powder was extracted with 500 μL of methanol:isopropanol:water (3:3:2, v/v/v), vortexed for 3 min, and sonicated at 4 °C for 30 min. The mixture was then centrifuged at 12,000 rpm for 3 min at 4 °C. Subsequently, 50 μL of supernatant was mixed with 20 μL of internal standard solution (1000 μg mL^−1^), evaporated under nitrogen, and freeze-dried. The residue was resuspended in 100 μL of methoxyamine hydrochloride in pyridine (15 mg mL^−1^) and incubated at 37 °C for 2 h, followed by the addition of 100 μL BSTFA and incubation at 37 °C for 30 min for derivatization. A 50 μL aliquot of the derivatized product was diluted to 1 mL with n-hexane and filtered through a 0.22 μm membrane prior to GC–MS analysis.

GC–MS analysis was performed using an Agilent 8890 gas chromatograph coupled to a 5977B mass spectrometer (Agilent, Santa Clara, CA, USA), equipped with a DB-5MS column (30 m × 0.25 mm × 0.25 μm). Helium was used as the carrier gas at a flow rate of 1 mL min^−1^. The injection volume was 1 μL, with a split ratio of 5:1. The oven temperature program was as follows: 160 °C for 1 min; increased to 200 °C at 6 °C min^−1^; increased to 270 °C at 10 °C min^−1^; increased to 300 °C at 5 °C min^−1^ and then increased to 320 °C at 20 °C min^−1^ and held for 5.5 min. The transfer line temperature was 280 °C, the ion source temperature was 230 °C, the quadrupole temperature was 150 °C, and the electron impact energy was 70 eV. Mass spectrometric acquisition was performed in selected ion monitoring (SIM) mode. Metabolites were quantified based on standard curves and internal standard correction. A pooled QC sample, prepared by mixing equal aliquots from all extracts, was injected after every 10 test samples to monitor instrument stability and analytical reproducibility. For metabolites quantified in the QC samples, the QC CVs were generally below 10% (Supplementary Table S15).

### 4.6. Analysis of Differential Metabolites

Metabolite content data were subjected to unit variance scaling (UV scaling) before clustering analysis and heatmap visualization. Differentially accumulated metabolites (DAMs) were identified through both pairwise and multi-group comparisons. For pairwise comparisons, DAMs were screened based on fold change (FC), using the criteria FC ≥ 2 or FC ≤ 0.5. DAMs identified by this criterion were used for statistical comparison among all pairwise comparison groups. For multi-group comparisons, OPLS-DA was performed, and differential metabolites were screened based on variable importance in projection (VIP) combined with one-way analysis of variance (ANOVA), using the criteria VIP > 1 and *p* < 0.05. In the present study, this analysis was mainly applied to overall differential metabolite comparisons among cultivars within the same tissue. Identified DAMs were further annotated based on the KEGG compound database and mapped to the KEGG pathway database for pathway classification and enrichment analysis, providing a basis for subsequent transcriptome–metabolome integrative analyses.

### 4.7. Integrative Analysis of the Transcriptome and Metabolome

To further integrate transcriptomic and metabolomic information, downstream integrative analyses were performed based on the standard transcriptomic and metabolomic results provided by Metware. First, KEGG analyses were conducted separately for DEGs and DAMs, and pathways to which both DEGs and DAMs were mapped within the same comparison group were defined as shared KEGG pathways. For each shared pathway, the numbers of pathway-mapped DEGs and DAMs were recorded, and the Benjamini–Hochberg-adjusted *p* values from DEG-based and DAM-based KEGG enrichment analyses were extracted. Recurrent shared pathways were summarized according to their detection frequency across the nine pairwise comparisons.

To evaluate the correspondence between gene expression changes and metabolite accumulation changes, Pearson correlation analysis was performed using gene expression and metabolite abundance data from all 18 samples. Significant DEG–DAM pairs were defined using the criteria |r| > 0.8 and *p* < 0.05. Based on these results, nine-quadrant plots were generated using the log_2_FC values of genes and metabolites in each comparison group, and DEG–DAM correlation heatmaps and network plots were constructed. For focused networks involving core candidate genes and candidate metabolites, a more stringent threshold of |r| ≥ 0.85 and *p* < 0.01 was applied.

In addition, canonical correlation analysis (CCA) and O2PLS were used for multivariate integration of the transcriptome and metabolome. CCA was performed separately for each shared KEGG pathway using the DEGs and DAMs within that pathway, whereas O2PLS was conducted based on the standardized quantitative matrix of all DEGs and DAMs to identify key transcripts and metabolites contributing strongly to the shared variation between the two omics datasets.

### 4.8. Weighted Gene Co-Expression Network Analysis (WGCNA)

Weighted gene co-expression network analysis was performed using the WGCNA package in R based on the normalized gene expression matrix of all 18 samples. Before network construction, genes showing consistently low expression (FPKM < 1 in more than 50% of samples) and low variance (coefficient of variation, *CV* ≤ 0.5) across all samples were filtered out using the varFilter function in the genefilter package to reduce background noise and improve network robustness. A pairwise adjacency matrix was calculated using Pearson correlation coefficients and raised to a soft-thresholding power β. To evaluate scale-free topology fit (R^2^) and mean connectivity, soft-thresholding powers (β = 1–20) were screened (Supplementary Figure S6). Driven by strong intrinsic transcriptomic heterogeneity across distinct cultivars and tissues, the signed R^2^ continuously increased with power without reaching the conventional threshold (R^2^ ≥ 0.85). In accordance with official WGCNA empirical guidelines for signed networks with sample sizes N < 20 (N = 18 in this study), β = 18 was selected as the soft-thresholding power. At this power, mean connectivity stabilized at a low level (≈ 15), guaranteeing network sparsity and minimizing correlation noise while preserving robust co-expression structures. The gene expression correlation matrix was then transformed into an adjacency matrix and further converted into a topological overlap matrix (TOM), followed by hierarchical clustering of genes. Co-expression modules were identified using the dynamic tree cut method, with the minimum module size (minModuleSize) set to 50. Modules with similar eigengene expression patterns were further merged, using a mergeCutHeight of 0.25.

For each co-expression module, the module eigengene was calculated to represent the overall expression pattern of the module. Pearson correlation analysis was then performed between module eigengenes and representative sugar metabolism-related traits, including the contents of glucose, fructose, maltose, and sucrose. Modules significantly correlated with these traits were regarded as candidate modules potentially associated with carbohydrate metabolic reprogramming and differential polysaccharide accumulation. To identify key candidate genes with high regulatory potential (hub genes) within target sugar-associated modules, a multi-parameter filtering strategy was implemented based on Module Membership (MM), Gene Significance (GS), and intramodular connectivity (k_Within_). Specifically, MM was calculated as the Pearson correlation between individual gene expression profiles and the module eigengene, reflecting the centrality of a gene within its assigned module. GS was defined as the absolute correlation between gene expression levels and specific soluble sugar traits. Genes meeting the criteria of high module belongingness (MM > 0.8), high trait association (|GS| > 0.6), and ranking within the top 10 of intramodular connectivity (k_Within_) were defined as core hub genes.

### 4.9. Statistical Analysis

Polysaccharide content data are presented as the mean ± standard deviation (SD) of three biological replicates. Crude polysaccharide content data were analyzed by two-way ANOVA using GraphPad Prism 9.0 to evaluate the effects of cultivar, tissue, and their interaction. Before ANOVA, normality of residuals and homogeneity of variance were assessed using residual diagnostics; no major violations of ANOVA assumptions were detected. When significant effects were found, post hoc multiple comparisons were performed using Sidak’s test for leaf-versus-stem comparisons within each cultivar and Tukey’s test for cultivar comparisons within each tissue. Statistical significance was set at *p* < 0.05.

For transcriptomic data, differential expression analysis was performed using DESeq2, and DEGs were identified using |log_2_FC| ≥ 1 and FDR < 0.05. For metabolomic data, pairwise comparisons used FC thresholds to screen differential candidate metabolites, whereas multi-group comparisons used VIP and ANOVA jointly. Correlation analyses were performed using Pearson correlation coefficients, with significance thresholds set to *p* < 0.05 or *p* < 0.01 depending on the specific analytical objective.

All statistical analyses and visualizations were conducted in the corresponding software environments. The main software and packages used in this study were as follows: fastp v0.23.2 for raw read quality control; HISAT2 v2.2.1 for read alignment; featureCounts v2.0.3 for gene-level read counting; StringTie v2.1.6 for transcript assembly and novel transcript prediction; DESeq2 v1.22.1 for differential expression analysis; clusterProfiler v4.6.0 for functional enrichment analysis [31]; WGCNA v1.72-1 for weighted gene co-expression network analysis [32]; genefilter v1.80.3 for expression matrix filtering; R v4.2.0 for statistical computing; pheatmap v1.0.10 and ComplexHeatmap v2.7.1.1009 for heatmap visualization; igraph v1.3.4 for network visualization; CCA v1.2.1 for canonical correlation analysis [33]; and OmicsPLS v2.0.2 for O2PLS analysis [34].

## 5. Conclusions

In conclusion, this study systematically revealed pronounced tissue- and cultivar-dependent differences in polysaccharide accumulation in *A. roxburghii* through the integration of polysaccharide quantification, transcriptomics, targeted sugar metabolomics, and multi-omics analyses. Stem tissues showed substantially higher polysaccharide contents than leaves, and cultivar-dependent variation was mainly manifested in stems, with JY exhibiting the strongest polysaccharide accumulation capacity. Multi-omics evidence consistently indicated that these differences were closely associated with coordinated carbohydrate metabolic reprogramming, particularly involving sucrose turnover, hexose redistribution, precursor sugar interconversion, nucleotide-sugar biosynthesis, and transport/allocation processes. Among the detected metabolites, glucose and D-fructose emerged as key hub nodes linking transcriptional variation to sugar metabolic differences. In addition, co-expression network analysis further identified candidate regulatory modules and genes potentially involved in precursor utilization, sugar partitioning, and polysaccharide-related metabolic regulation. Overall, this study provides new insights into the molecular basis of polysaccharide accumulation in *A. roxburghii* and offers a theoretical foundation for elite germplasm screening, quality evaluation, and precision cultivation of high-polysaccharide materials.

## Figures and Tables

**Table 1:** High-frequency sugar-related differentially accumulated metabolites detected in different pairwise comparison groups.

Abbreviation	Metabolite	Frequency	Regulation Pattern	Fold Change Range
Fru	D-Fructose	8	8 up, 0 down	2.49–21.05
Xyl	D-Xylose	8	7 up, 1 down	0.28–24.17
Glu	Glucose	6	5 up, 1 down	0.39–5.50
Mal	Maltose	4	4 up, 0 down	Inf
Ino	Inositol	3	3 up, 0 down	2.35–4.14
D-Ara	D-Arabinose	3	2 up, 1 down	2.35–4.14

Frequency indicates the number of pairwise comparison groups in which each sugar-related differentially accumulated metabolite was detected. Fold change range and regulation pattern were summarized from Supplementary Table S3. Positive log_2_FC values indicate higher accumulation in the first group of the comparison, whereas negative log_2_FC values indicate lower accumulation in the first group. “Up” and “down” indicate up- or down-accumulation in the first group relative to the second group. “Inf” indicates that the metabolite was detected in one group but not the other, resulting in an infinite fold change. Detailed pairwise comparison-specific fold changes, log_2_FC values and regulation directions are provided in Supplementary Table S3. DAMs in pairwise comparisons were screened using the criteria FC ≥ 2 or FC ≤ 0.5. Only representative sugar-related DAMs detected repeatedly in at least three comparison groups are listed.

**Table 2:** Representative recurrent shared KEGG pathways associated with differential polysaccharide accumulation in *A. roxburghii*.

Pathway	Joint Mapping Frequency	DEG No. Range	BH-Adjusted *p* Value for DEG Enrichment	DAM No. Range	BH-Adjusted *p* Value for DAM Enrichment	Biological Relevance
Starch and sucrose metabolism (ko00500)	9/9	239–312	2.54 × 10^−30^–1.000	1–4	0.0196–1.000	Sucrose turnover and hexose supply.
Amino sugar and nucleotide sugar metabolism (ko00520)	9/9	145–213	3.25 × 10^−18^–0.2407	2–4	0.4118–1.000	Activated sugar-intermediate supply.
Biosynthesis of nucleotide sugars (ko01250)	9/9	96–157	1.47 × 10^−10^–0.3929	1–3	1	UDP-/GDP-sugar donor formation.
Fructose and mannose metabolism (ko00051)	9/9	64–109	1.60 × 10^−7^–0.6775	1–2	1	Precursor interconversion and carbon allocation.
Galactose metabolism (ko00052)	9/9	45–102	1.93 × 10^−6^–1.000	1–4	1	Soluble-sugar remodeling and precursor supply.
Pentose and glucuronate interconversions (ko00040)	8/9	122–172	4.89 × 10^−21^–1.000	1–2	1	Pentose- and uronic acid-precursor formation.

Frequency indicates the number of pairwise comparison groups in which the corresponding pathway was detected as a shared KEGG pathway in the integrated transcriptome–metabolome analysis. Pathway-mapped DEG and DAM numbers indicate the numbers of DEGs and DAMs assigned to the corresponding KEGG pathway in each comparison group. The adjusted *p* values represent Benjamini–Hochberg-corrected *p* values from DEG-based or DAM-based KEGG enrichment analyses, respectively. Values are shown as ranges across the corresponding comparison groups. Detailed comparison-specific DEG/DAM numbers and adjusted *p* values are provided in Supplementary Table S6B. Representative high-frequency pathways with relatively direct mechanistic relevance to sugar source supply, precursor sugar conversion, or nucleotide sugar donor formation are prioritized here; other recurrent shared pathways are listed in Supplementary Table S6A.

## Data Availability

The raw RNA-Seq data reported in this paper have been deposited in the National Genomics Data Center (NGDC) under BioProject accession number PRJCA069658. All other data supporting the findings of this study are available within the article and its Supplementary Materials.

## Supplementary Materials

The following supporting information can be downloaded at https://www.mdpi.com/article/10.3390/ijms27156917/s1.
