# Supplementary material for: Transcriptomic and Metabolomic Insights into Tissue- and Cultivar-Dependent Polysaccharide Accumulation in Anoectochilus roxburghii
Source: Int J Mol Sci. 2026 Aug 1;27(15):6917. doi: 10.3390/ijms27156917 (PMC13467593; doi:10.3390/ijms27156917)
Supplement: Supplementary file 1 [file ijms-27-06917-s001.zip › Supplementary Figures.pdf]

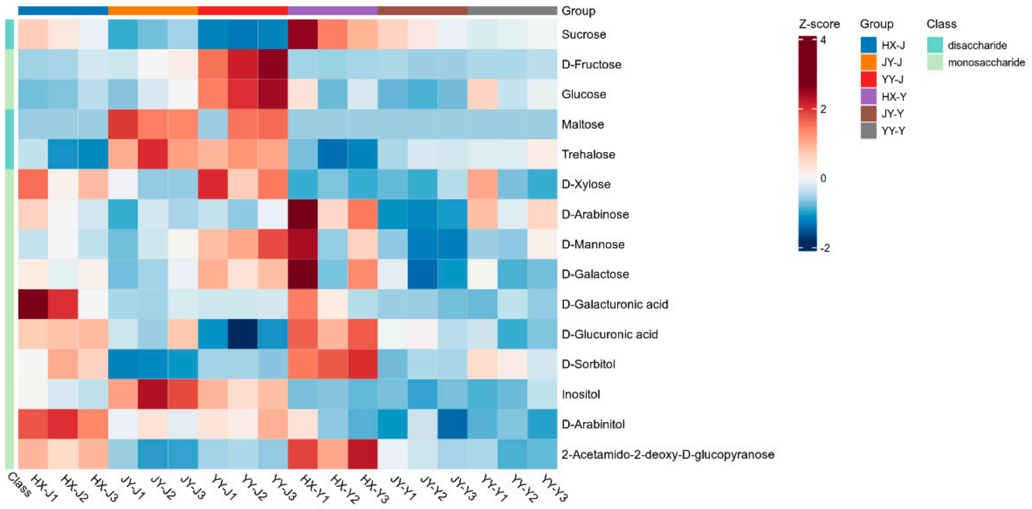

Supplementary Figure S1. Heatmap showing the relative accumulation patterns of all identified differentially accumulated metabolites across cultivars and tissues

This heatmap shows the relative accumulation patterns of all differentially accumulated metabolites (DAMs) across 18 samples from three varieties of Anoectochilus and two tissues. The colors represent row-normalized Z-score values, with red indicating relatively high accumulation and blue indicating relatively low accumulation. The samples are arranged in order by tissue and variety. This figure provides the complete information on the variation patterns of all DAMs, beyond the representative DAMs shown in Figure 3 of the main text.

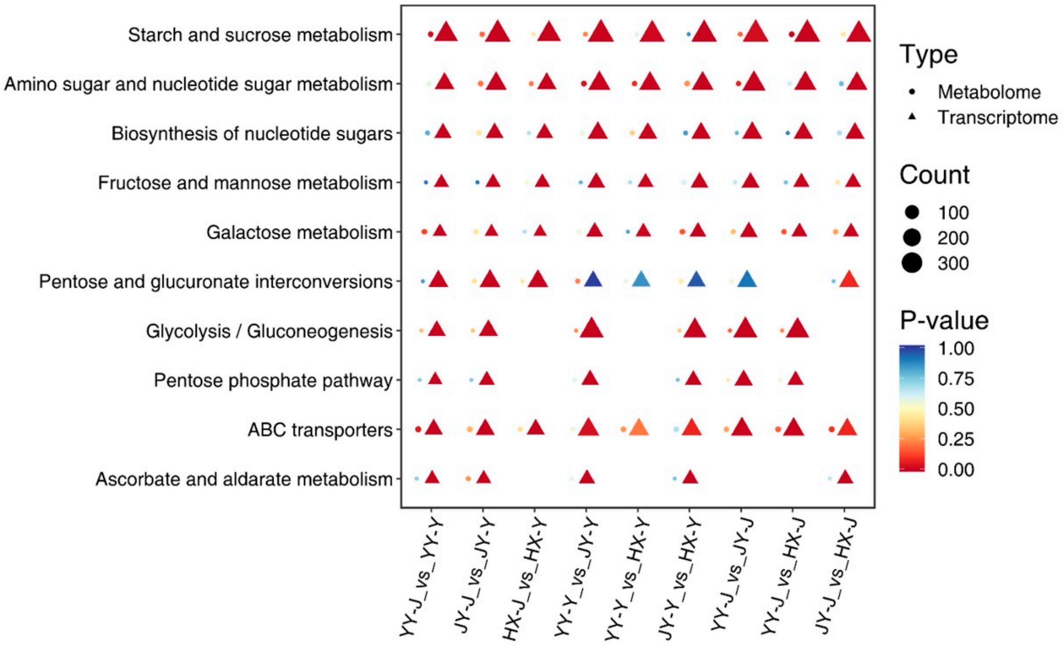

Supplementary Figure S2. Distribution of frequently co-occurring KEGG pathways across nine pairwise comparison groups.

Each column represents one pairwise comparison group, and each row represents a KEGG pathway that appears repeatedly in multiple comparison groups. Circles and triangles indicate enrichment results in the metabolome and transcriptome, respectively, for the corresponding comparison group. The size of the symbols represents the number of hits, and the color indicates the significance level of enrichment. This figure illustrates the distribution patterns of high-frequency pathways at both transcriptomic and metabolomic levels across comparisons of different varieties and tissues.

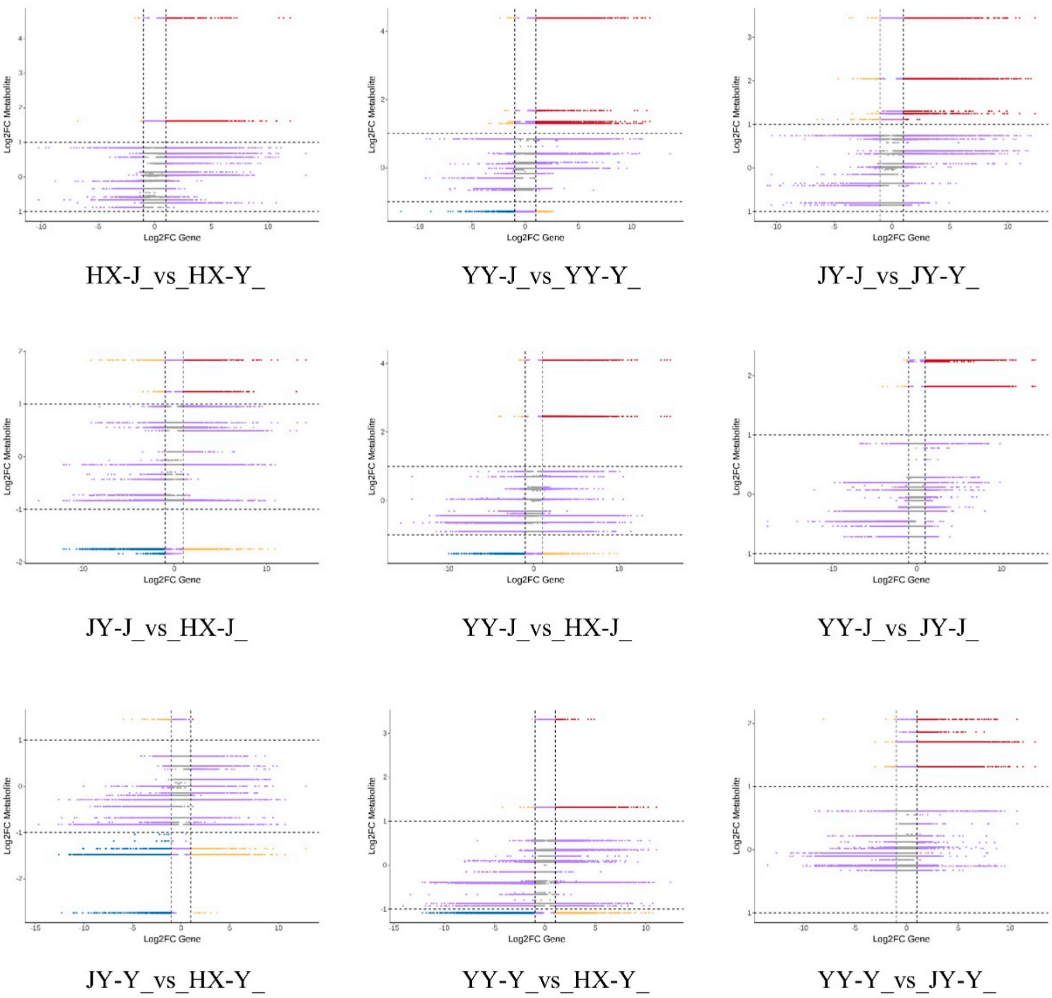

Supplementary Figure S3. Nine-quadrant distribution of significant DEG–DAM pairs across all pairwise comparisons in *A. roxburghii.*

Nine-quadrant plots were generated based on Pearson correlation analysis between DEGs and DAMs across all 18 samples. Significant DEG–DAM pairs were defined as $|r| > 0.8$ and $P < 0.05$. Each point represents one significant DEG–DAM pair. The x-axis indicates gene log2FC, and the y-axis indicates metabolite log2FC in the corresponding comparison group. Dashed lines indicate the thresholds for differential gene expression and differential metabolite accumulation (log2FC = ±1). The upper-right and lower-left regions represent coordinated upregulation and coordinated downregulation of genes and metabolites, respectively, whereas the upper-left and lower-right regions indicate opposite directional changes. The plots summarize the distribution patterns of significant DEG–DAM pairs across the nine pairwise comparisons and support the assessment of coordinated transcriptomic and metabolic variation among tissues and cultivars.

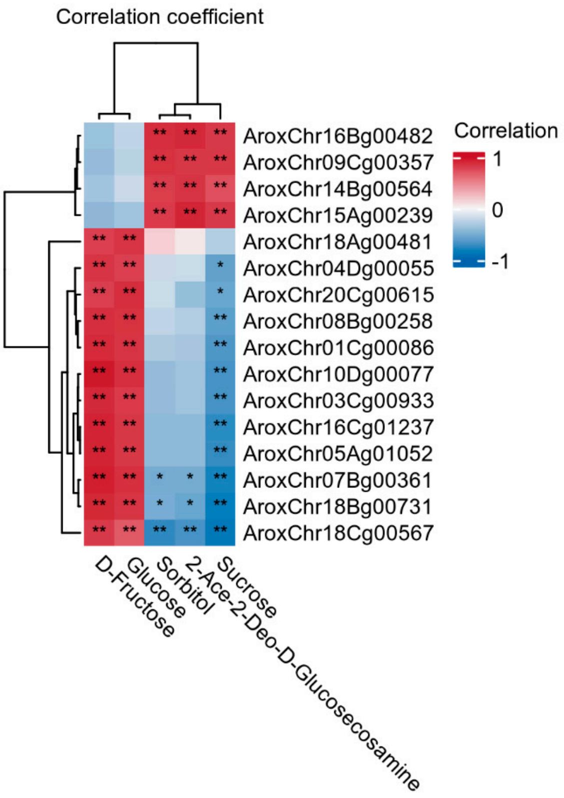

Supplementary Figure S4. Pearson correlation heatmap of candidate genes and key metabolites associated with differential polysaccharide accumulation.

The heatmap shows the Pearson correlation coefficients between 16 candidate genes and 5 key metabolites across all 18 samples. Red and blue indicate positive and negative correlations, respectively, with color intensity representing the magnitude of the correlation coefficient. Both rows and columns are hierarchically clustered according to correlation patterns. * and ** denote $P < 0.05$ and $P < 0.01$, respectively. The candidate genes and metabolites correspond to the nodes used in the key correlation network shown in Figure 4C.

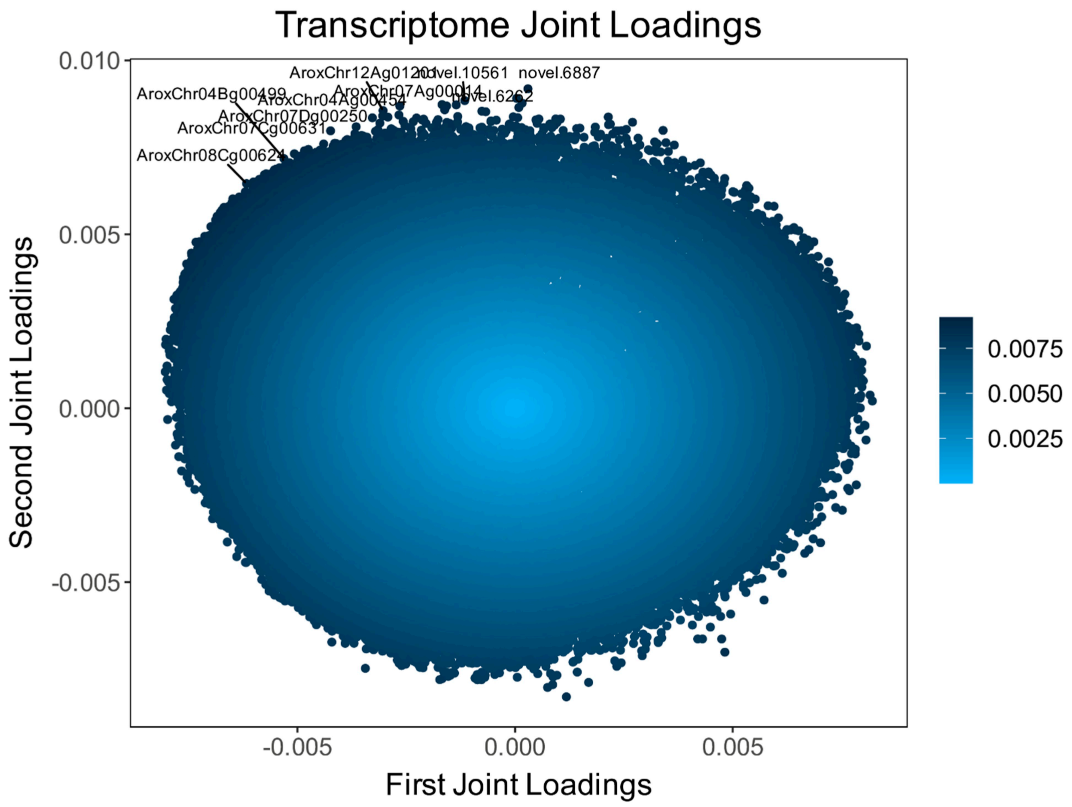

Supplementary Figure S5. O2PLS transcriptome joint loadings associated with transcriptome–metabolome covariation in *A. roxburghii.*

Each point represents one transcript variable projected into the O2PLS joint loading space based on the standardized DEG and DAM matrices. The first and second joint loadings reflect the contribution patterns of transcript variables to the shared variation between transcriptomic and sugar metabolomic datasets. Most transcript variables were concentrated near the center of the plot, indicating that individual transcript contributions to the joint transcriptome–metabolome structure were generally dispersed. Only representative transcripts with relatively high joint loading values are labeled, including novel.6887, AroxChr07Ag00014, AroxChr12Ag01201, AroxChr07Cg00631, AroxChr07Dg00250, AroxChr04Ag00454, and AroxChr08Cg00624. These high-loading transcripts may represent candidate variables associated with sugar metabolic remodeling and differential polysaccharide accumulation in *A. roxburghii*, but their specific functions require further experimental validation.

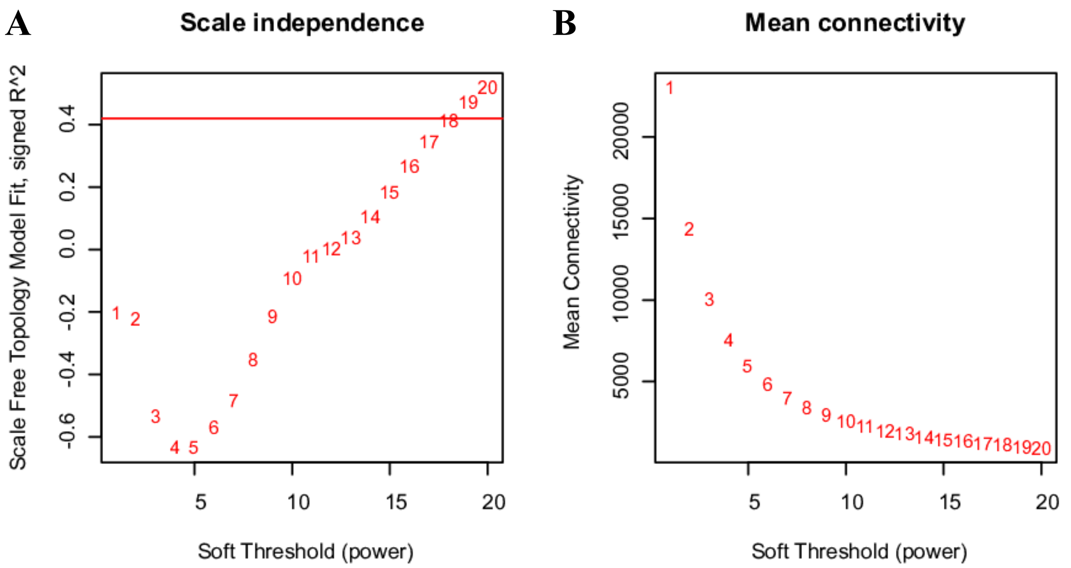

Supplementary Figure S6. Determination of the soft-thresholding power (β) for weighted gene co-expression network analysis (WGCNA).

(A) Analysis of the scale-free fit index (signed R^2) as a function of soft-thresholding powers ranging from 1 to 20. The red horizontal line marks the selected soft-thresholding power (β = 18). (B) Analysis of mean connectivity as a function of soft-thresholding powers. Power β = 18 was selected in accordance with WGCNA empirical guidelines for signed networks (N = 18) to balance network sparsity and scale-free topology approximation across heterogeneous cultivar and tissue samples.

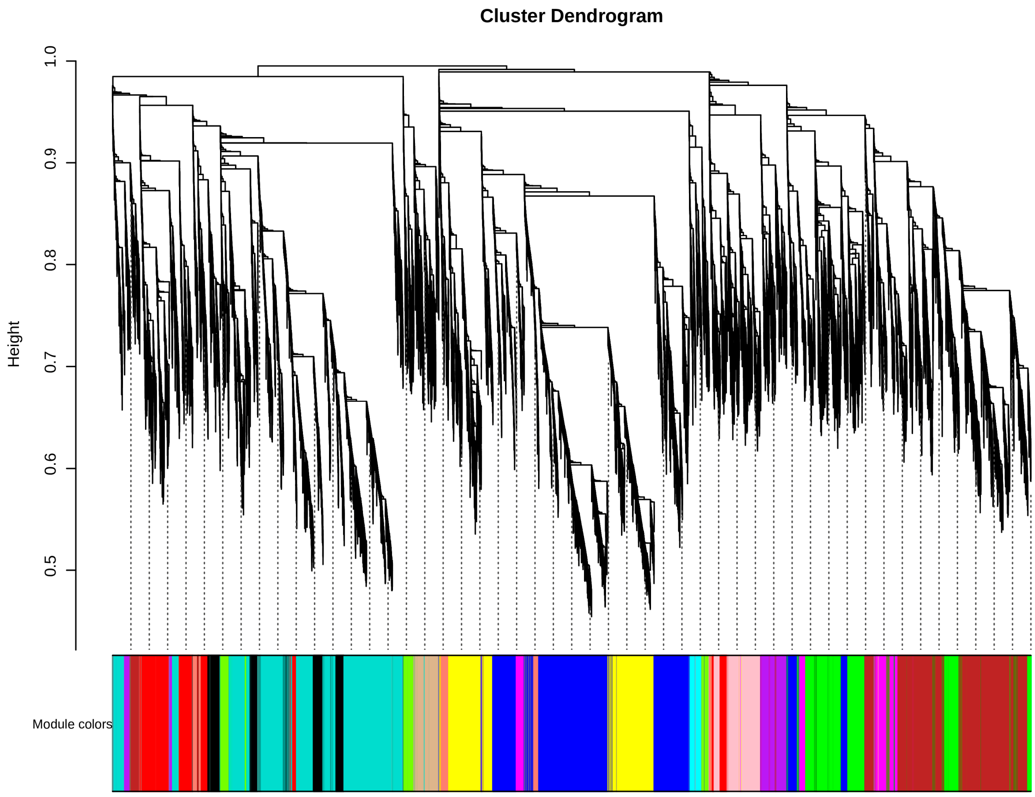

Supplementary Figure S7. Hierarchical clustering dendrogram of co-expressed genes and co-expression module identification.

Each color assigned beneath the dendrogram represents a distinct co-expression module comprising genes with similar expression profiles across various cultivars and tissues, indicating potential functional associations. In the upper dendrogram, the vertical distance (height) represents the dissimilarity between genes (nodes), whereas the horizontal distance carries no mathematical or biological significance.

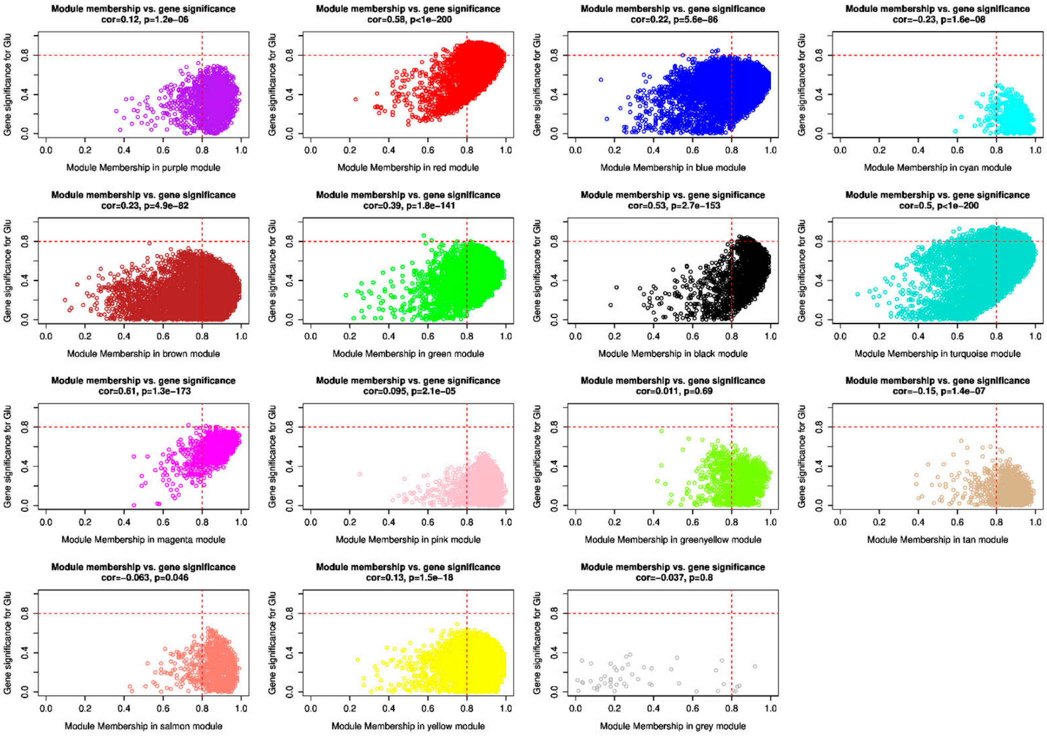

Supplementary Figure S8. Scatter plots of Module Membership (MM) versus Gene Significance (GS) for glucose content across co-expression modules.

Each node represents an individual gene within the designated module. The x-axis indicates the MM (kME), quantifying the intramodular connectivity of a gene, and the y-axis represents the GS, measuring the correlation between gene expression and glucose (Glu) accumulation. Pearson correlation coefficients (*cor*) and *p*-values (*p*) are displayed at the top of each subplot.

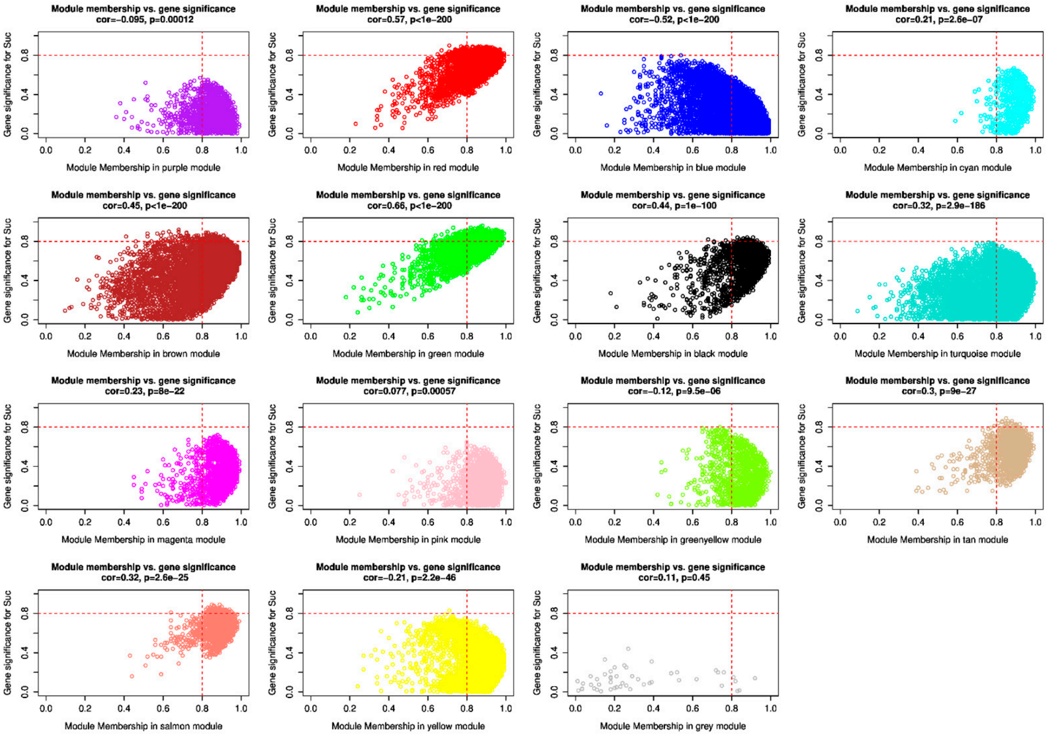

Supplementary Figure S9. Scatter plots of Module Membership (MM) versus Gene Significance (GS) for sucrose content across co-expression modules.

Each node represents an individual gene within the designated module. The x-axis indicates the MM (kME), quantifying the intramodular connectivity of a gene, and the y-axis represents the GS, measuring the correlation between gene expression and sucrose (Suc) accumulation. Pearson correlation coefficients (*cor*) and *p*-values (*p*) are displayed at the top of each subplot.

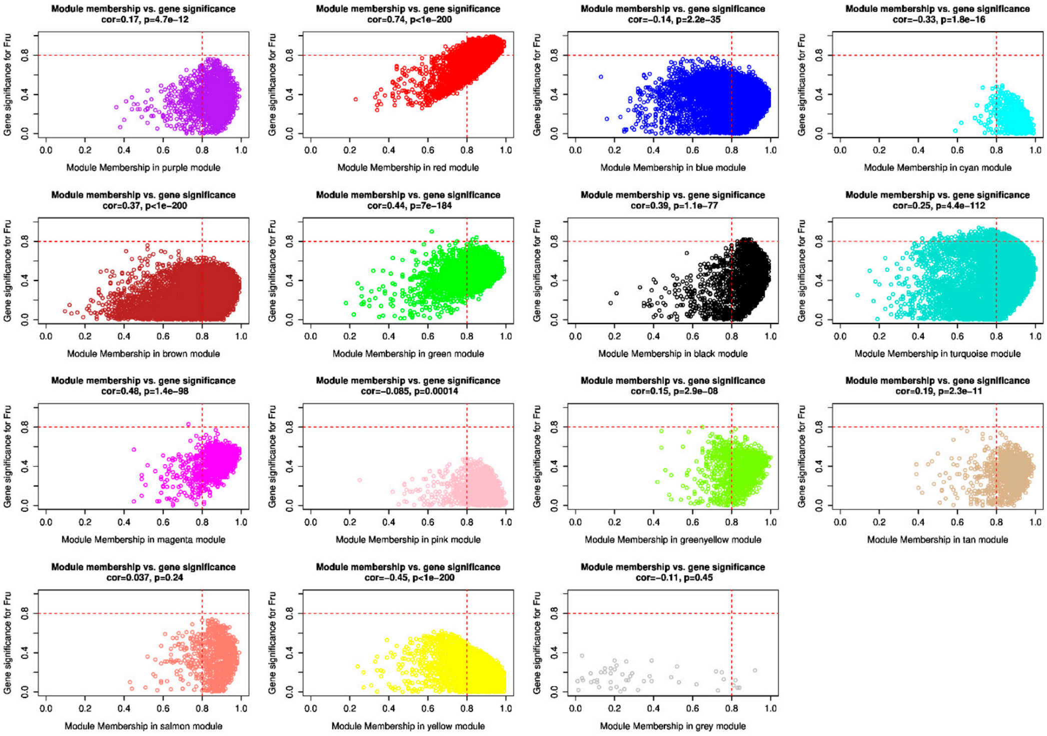

Supplementary Figure S10. Scatter plots of Module Membership (MM) versus Gene Significance (GS) for fructose content across co-expression modules.

Each node represents an individual gene within the designated module. The x-axis indicates the MM (kME), quantifying the intramodular connectivity of a gene, and the y-axis represents the GS, measuring the correlation between gene expression and fructose (Fru) accumulation. Pearson correlation coefficients (*cor*) and *p*-values (*p*) are displayed at the top of each subplot.

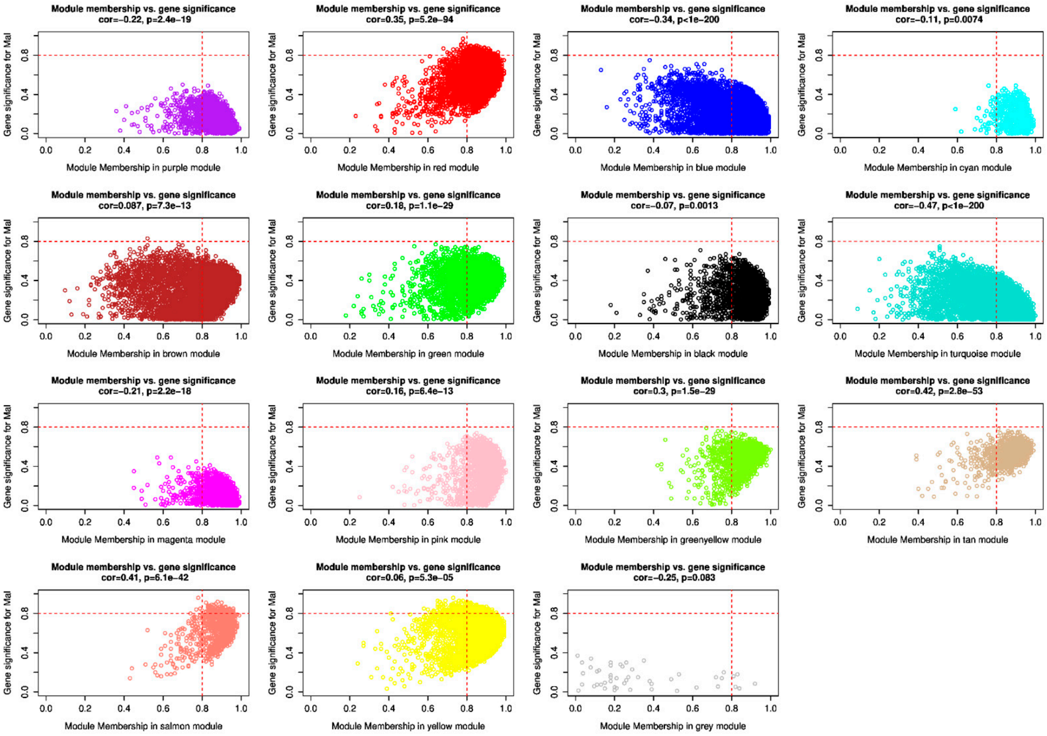

Supplementary Figure S11. Scatter plots of Module Membership (MM) versus Gene Significance (GS) for maltose content across co-expression modules.

Each node represents an individual gene within the designated module. The x-axis indicates the MM (kME), quantifying the intramodular connectivity of a gene, and the y-axis represents the GS, measuring the correlation between gene expression and maltose (Mal) accumulation. Pearson correlation coefficients (*cor*) and *p*-values (*p*) are displayed at the top of each subplot.
